# Microarray screening reveals two non-conventional SUMO-binding modules linked to DNA repair by non-homologous end-joining

**DOI:** 10.1093/nar/gkac237

**Published:** 2022-04-14

**Authors:** Maria Jose Cabello-Lobato, Matthew Jenner, Metztli Cisneros-Aguirre, Kira Brüninghoff, Zac Sandy, Isabelle C da Costa, Thomas A Jowitt, Christian M Loch, Stephen P Jackson, Qian Wu, Henning D Mootz, Jeremy M Stark, Matthew J Cliff, Christine K Schmidt

**Affiliations:** Manchester Cancer Research Centre (MCRC), Division of Cancer Sciences, School of Medical Sciences, Faculty of Biology, Medicine and Health, University of Manchester, 555 Wilmslow Road, Manchester M20 4GJ, UK; Department of Chemistry, University of Warwick, Coventry CV4 7AL, UK; Warwick Integrative Synthetic Biology (WISB) Centre, University of Warwick, Coventry CV4 7AL, UK; Department of Cancer Genetics and Epigenetics, Irell and Manella Graduate School of Biological Sciences Beckman Research Institute of the City of Hope, 1500 E Duarte Rd, Duarte, CA 91010, USA; Institute of Biochemistry, University of Muenster, Corrensstraße 36, 48149 Muenster, Germany; Manchester Cancer Research Centre (MCRC), Division of Cancer Sciences, School of Medical Sciences, Faculty of Biology, Medicine and Health, University of Manchester, 555 Wilmslow Road, Manchester M20 4GJ, UK; Manchester Cancer Research Centre (MCRC), Division of Cancer Sciences, School of Medical Sciences, Faculty of Biology, Medicine and Health, University of Manchester, 555 Wilmslow Road, Manchester M20 4GJ, UK; Wellcome Trust Centre for Cell-Matrix Research, School of Biological Sciences, Faculty of Biology, Medicine and Health, University of Manchester, Oxford Road, Manchester M13 9PT, UK; AVM Biomed, Pottstown, PA19464, USA; Wellcome/Cancer Research UK Gurdon Institute and Department of Biochemistry, University of Cambridge, Cambridge CB2 1QN, UK; Astbury Centre for Structural Molecular Biology, School of Molecular & Cellular Biology, Faculty of Biological Sciences, University of Leeds, Leeds LS2 9JT, UK; Institute of Biochemistry, University of Muenster, Corrensstraße 36, 48149 Muenster, Germany; Department of Cancer Genetics and Epigenetics, Irell and Manella Graduate School of Biological Sciences Beckman Research Institute of the City of Hope, 1500 E Duarte Rd, Duarte, CA 91010, USA; Manchester Institute of Biotechnology (MIB) and School of Chemistry, University of Manchester, Manchester M1 7DN, UK; Manchester Cancer Research Centre (MCRC), Division of Cancer Sciences, School of Medical Sciences, Faculty of Biology, Medicine and Health, University of Manchester, 555 Wilmslow Road, Manchester M20 4GJ, UK

## Abstract

SUMOylation is critical for numerous cellular signalling pathways, including the maintenance of genome integrity via the repair of DNA double-strand breaks (DSBs). If misrepaired, DSBs can lead to cancer, neurodegeneration, immunodeficiency and premature ageing. Using systematic human proteome microarray screening combined with widely applicable carbene footprinting, genetic code expansion and high-resolution structural profiling, we define two non-conventional and topology-selective SUMO2-binding regions on XRCC4, a DNA repair protein important for DSB repair by non-homologous end-joining (NHEJ). Mechanistically, the interaction of SUMO2 and XRCC4 is incompatible with XRCC4 binding to three other proteins important for NHEJ-mediated DSB repair. These findings are consistent with SUMO2 forming a redundant NHEJ layer with the potential to regulate different NHEJ complexes at distinct levels including, but not limited to, XRCC4 interactions with XLF, LIG4 and IFFO1. Regulation of NHEJ is not only relevant for carcinogenesis, but also for the design of precision anti-cancer medicines and the optimisation of CRISPR/Cas9-based gene editing. In addition to providing molecular insights into NHEJ, this work uncovers a conserved SUMO-binding module and provides a rich resource on direct SUMO binders exploitable towards uncovering SUMOylation pathways in a wide array of cellular processes.

## INTRODUCTION

Posttranslational modification (PTM) with SUMO (small ubiquitin-like modifier) is key to regulating a panoply of cellular signalling pathways, including transcription, chromatin organisation, nuclear trafficking, DNA replication and DNA repair (1,2). It is therefore not surprising that deregulation of the SUMO system is associated with a range of prevalent human diseases including neurodegenerative disorders, cardiovascular diseases and cancer (1). In humans, the SUMO paralogues SUMO1-3 are ubiquitously expressed and established as posttranslational modifiers. SUMO2 and SUMO3 are almost identical, sharing 97% sequence identity, whereas SUMO1 and SUMO2/3 are ∼55% different. SUMOylation is mediated by an enzymatic triad: an activating E1 enzyme – a heterodimer formed by UBA2 (aka SAE2) and SAE1, the conjugating E2 enzyme UBE2I (aka UBC9), and one of ∼10 E3 ligases. SUMOylation can occur on one or multiple lysines of substrate proteins as monomers or chains of multiple SUMO moieties, creating a complex SUMO code. PolySUMO chains in cells are primarily formed by SUMO2/3 linked via their internal K11 residues, with SUMO1 mainly being deemed a chain terminator. Biochemical outcomes for distinct SUMO architectures can differ, with polySUMO chains being formed particularly in response to certain types of stressors, suggesting their importance in responding to such stimuli. However, we still know little about how polySUMO chains regulate specific cell signalling events (3). By translating SUMOylations into defined biochemical actions, SUMO receptors – proteins non-covalently binding to and recognising SUMO topologies – play key roles in determining the functional outcomes of SUMOylation events. Despite their importance and the large number (>7000) of substrate SUMOylations existing in human cells, only few (several tens) of SUMO receptors have been validated, and even less have been characterised for their binding to different SUMO topologies. As a consequence, little is known about length- and paralogue-selective recognition of SUMO topologies. Indeed, knowledge of different SUMO-binding modules is mostly limited to a small number of varying themes centred on 4–5 hydrophobic amino acids called SUMO interacting motifs (SIMs) (4,5,6). This knowledge-gap limits our understanding of how SUMO functions at mechanistic levels and how it can best be exploited for treating human diseases associated with SUMO dysfunctions.

Here, we systematically screen the human proteome for receptors of polySUMO2 chains, identifying hundreds of candidates with diverse roles in established and emerging areas of SUMO biology. We validate a substantial and functionally varied set of SUMO receptors followed by in-depth characterisation of the SUMO-binding modules of one of the identified receptors, XRCC4. XRCC4 is a core DNA repair factor known for its importance in DNA double-strand break (DSB) repair by non-homologous end-joining (NHEJ). DNA damage occurs frequently and can be caused by endogenous and exogenous sources. DSBs are the most cytotoxic DNA lesions and if left mis- or unrepaired, they can lead to cell death, mutagenesis or chromosomal translocation, and in turn cancer (7). Cells have evolved two major pathways to repair DSBs: homologous recombination (HR), which repairs DSBs with high fidelity in late S/G2 cell cycle phases using a homologous sequence as a template, usually the sister chromatid; and NHEJ, which is less accurate than HR, but functions throughout interphase and repairs the large majority of DSBs in mammalian cells (8). The importance for, and underlying mechanisms of, the SUMO system for key aspects of DSB repair by HR are well established, with SUMOylations of various HR factors and their decoding mechanisms via downstream receptors characterised (9). By contrast, little is known about how SUMOylation regulates NHEJ. Here, we identify and characterise two distinct non-conventional polySUMO2-binding modules on XRCC4 located in its head and coiled-coil domains and demonstrate the relevance of one of them for DNA repair by NHEJ, as well as for our understanding of SUMO-binding in other, non-DNA repair-related proteins. Due to their locations, XRCC4 interactions with SUMO2 represent prime targets for regulating NHEJ at the level of three other key NHEJ factors, XLF, LIG4 and IFFO1, and/or via XRCC4 complexes functioning independently of these proteins (10).

## MATERIALS AND METHODS

### PolySUMO2 microarray staining and analysis

PolySUMO2 chains (#ULC-220, Boston Biochem) were directly labeled with Cy5 following the manufacturer's guidelines (GE Healthcare). After 45 min incubation in the dark, 10% reaction volume of 2 M Tris–HCl pH 7.5 were added to quench unreacted dye, and the incubation extended in the dark for 10 min. PolySUMO2 chains were then purified in PD25 spin columns according to the manufacturer's recommendation (GE Healthcare). The purified and labeled polySUMO2 chains were immediately applied to blocked human proteome microarrays (HuProt™v2.0, CDI Laboratories) that contain >21 000 protein spots, representing ∼15 000 unique proteins with one or more isoforms. Microarrays were removed from −20°C storage and placed at room temperature (RT) for 15 min before opening, to avoid condensation. Arrays were then blocked for 1 h at RT in PBS containing 0.05% Tween-20, 20 mM reduced glutathione, 1 mM DTT, 3% BSA, and 25% glycerol. Three PBS washes preceded a 90 min incubation step at RT with labelled polySUMO2 chains (or Tris-quenched Cy5 dye as a reference). After two washing steps with PBS containing 0.05% Tween-20, two PBS washes, and two washes with water, centrifugal drying (1000 rpm for 5 min at RT) was performed and the arrays scanned using a GenePix scanner (4100A by Molecular Devices). Microarray images were gridded and quantitated using GenePix Pro (v7) software. Median intensities (features and local backgrounds) were utilised, and signal-to-noise ratios calculated. Values were then normalised to biological controls within each array and duplicate features (representing identical proteins) summarised by average. These values were compared between arrays (polySUMO2-bound minus mock-treated array) then Loess transformed by print tip and location to remove technical sources of error (11), resulting in the final estimate of magnitude change (*M*-value). The threshold for proteins classifying as polySUMO2 receptor candidates was set to 1 standard deviation of the population above the population average. Given that the *M*-value is a twice-normalised (biologically and for technical sources of error) difference between mean signal-to-noise ratios generated from relative fluorescence units, it is reported/graphed as ‘*M*-value’ without units.

### Carbene footprinting

Samples were prepared and analysed as previously described (12). Briefly, 20 μM full-length (FL) XRCC4 or 25 μM XRCC4^1–164^ were mixed with 20 or 25 μM of mSUMO2, respectively, in a buffer containing 20 mM HEPES pH 6.8, 140 mM NaCl, 1 mM EDTA, 2 mM DTT and 0.02% NaN_3_, as well as 10 mM of aryldiazirine probe (total volume, 20 μl). The mixture was left to equilibrate for 5 min at RT before 6 μl aliquots were placed in crystal-clear vials (Fisher Scientific UK) and snap-frozen in liquid nitrogen. The labelling reaction was initiated by photolysis of the mixture using the third harmonic of a Nd:YLF laser (Spectra Physics, repetition frequency 1000 Hz, pulse energy 125 μJ) at a wavelength of 347 nm. The frozen samples were irradiated for 10 s. All experiments were performed in triplicate. Following irradiation, samples were thawed, reduced (10 mM DTT in 10 mM ammonium bicarbonate), alkylated (55 mM iodoacetamide in 10 mM ammonium bicarbonate) and incubated at 37°C with trypsin overnight (1:20 protease/protein ratio in 10 mM ammonium bicarbonate). The analysis of the digests was carried out on a Bruker MaXis II ESI-Q-TOF-MS connected to a Dionex 3000 RS UHPLC fitted with an ACE C18 RP column (100 × 2.1 mm, 5 μm, 30°C). The column was eluted with a linear gradient of 5–100% MeCN containing 0.1% formic acid over 40 min. The mass spectrometer was operated in positive ion mode with a scan range of 200–3000 *m*/*z*. Source conditions were: end plate offset at −500 V; capillary at −4500 V; nebulizer gas (N_2_) at 1.6 bar; dry gas (N_2_) at 8 l/min; dry temperature at 180°C. Ion transfer conditions were: ion funnel RF at 200 V_pp_; multiple RF at 200 V_pp_; quadrupole low mass at 55 *m*/*z*; collision energy at 5.0 eV; collision RF at 600 V_pp_; ion cooler RF at 50–350 V_pp_; transfer time at 121 s; pre-pulse storage time at 1 μs. A previously described method was used to quantitate the fraction of each peptide modified (13). Briefly, the chromatograms for each singly-labelled and unlabelled peptide were extracted within a range of ±0.1 *m*/*z* and the spectrum for each peak was manually inspected to ensure the sampling of the correct ion only. The peptide fractional modification was calculated using Equation 1 below, where A(labelled) and A(unlabelled) correspond respectively to the peak area of each labelled and unlabelled peptide. Differences in the extent of labelling between peptides were considered significant when the *P* value obtained from a Student *t*-test was <0.05.

(1) $$\begin{equation*}P\ = \ \frac{{A\left( {{\rm labelled}} \right)}}{{A\left( {{\rm labelled}} \right) + A\left( {{\rm unlabelled}} \right)}}\end{equation*}$$

### NMR spectra acquisition and analysis

Protein spectra were recorded at 310 K on a Bruker 800 MHz spectrometer with a ^1^H/^13^C–^15^N TCI cryoprobe equipped with z-gradients in 20 mM HEPES pH 6.8, 140 mM NaCl, 1 mM EDTA, 2 mM DTT, 100 mM arginine, 100 mM glutamic acid, 0.02% NaN_3_, unless otherwise specified. XRCC4 ^1^H–^15^N spectra were standard Bruker BEST-TROSY with phase-sensitive Echo/Antiecho gradient selection. SUMO ^1^H–^15^N spectra were standard Bruker sensitivity-enhanced, phase-sensitive HSQC spectra using Echo/Antiecho gradient selection. 1D ^1^H spectra were recorded using excitation sculpting water suppression. The assignments for mSUMO1 and mSUMO2 were taken from BMRB entries 25576 and 6801, respectively, and temperature and buffer conditions were incremented from the conditions used in the assignments to those used for this study, to allow for the associated chemical shift changes. Assignment of 6×His-XRCC4^1–164^ was carried out as described (14). Assignment of 6×His-XRCC4^1–180^ was attempted by the same methods using a ^1^H–^15^N–^13^C-labelled protein sample. The majority of ^1^H^15^N crosspeaks were extremely low intensity, but in the same positions as in the 6×His-XRCC4^1–164^ construct. Residues 167–180 were assigned. Assignments are deposited with BMRB code 50742. Data were processed and visualised using Topspin 3.5 (Bruker), and protein backbone assignment was done using CCPN Analysis 2.1. Protein:protein interactions were analysed using CCPNAssign (v3.1).

### Colour gradients for NMR analyses

#### ^15^N-labelled XRCC4^1–164^ plus mSUMO2

Colour gradient range for the XRCC4 structure (PDB 1IK9) and sequence was based on intensity losses in the ^1^H–^15^N BEST-TROSY spectra of XRCC4^1–164^ after addition of increasing concentrations of mSUMO2 as follows: from red (most affected by binding: <45% intensity after addition of 0.1 monomer equivalents) to blue (unaffected by 4-fold excess over monomer), with intermediate points as follows: 45–66% intensity at 0.1 equivalents, <45% intensity at 0.5 equivalents, 45–66% intensity at 0.5 equivalents, <45% intensity at 1 equivalent (white), 45–66% intensity at 1 equivalent, <45% intensity at 4 equivalents and 45–66% intensity at 4 equivalents.

#### ^15^N-labelled XRCC4^1–164^ plus diSUMO2

Colour gradient range for XRCC4 structure (PDB 1IK9) and sequence was based on intensity losses in the ^1^H–^15^N BEST-TROSY spectra of XRCC4^1–164^ after addition of increasing concentrations of diSUMO2 as follows: from red (most affected by binding: <20% intensity after addition of 0.125 monomer equivalents), to blue (unaffected by 0.25 equivalents), with intermediate points as follows: 20–30% intensity, 30–45% intensity, 45–66% intensity at 0.125 equivalents; <20% intensity (white), 20–30% intensity, 30–45% and 45–66% intensity at 0.25 equivalents.

#### ^15^N-labelled XRCC4^1–164^ plus 4×SUMO2

Colour gradient range for XRCC4 structure (PDB 1IK9) and sequence was based on intensity losses in the ^1^H–^15^N BEST-TROSY spectra of XRCC4^1–164^ after addition of increasing concentrations of 4×SUMO2 as follows: from red (most affected by binding: <20% intensity on addition of 0.1 monomer equivalents) to blue (unaffected by 0.67 equivalents), with intermediate points as follows: 20–30% intensity, 30–45% intensity and 45–66% intensity at 0.1 equivalents; <20% intensity (white), 20–30% intensity, 30–45% and 45–66% intensity at 0.67 equivalents.

#### ^15^N-labelled XRCC4^1–164^ plus mSUMO1

Colour gradient range for XRCC4 structure (PDB 1IK9) and sequence was according to chemical shift perturbations (CSP) in the ^1^H–^15^N-TROSY NMR spectra of XRCC4^1–164^ after addition of 1 monomer equivalent of mSUMO1 as follows: from red (most affected by binding: >5 standard deviations intensity on addition of 1 monomer equivalents), to blue (unaffected by 1 equivalent), with intermediate points as follows: 4, 3, 2 and 1 standard deviation(s).

#### ^15^N-labelled mSUMO2 plus XRCC4^1–164^

Colour gradient range for mSUMO2 structure (PDB 2N1W) and sequence was based on intensity losses in the ^1^H–^15^N HSQC spectra of mSUMO2 after addition of increasing concentrations of XRCC4^1–164^ as follows: from red (most affected by binding: <20% intensity after addition of 0.25 mSUMO2 equivalents) to blue (unaffected by 1 equivalent), with intermediate points as follows: 21–30% intensity, <31–40% intensity and 41–66% intensity at 0.25 equivalents; <20% intensity (white), 21–30% intensity, 31–40% intensity and 41–66% intensity at 0.5 equivalents.

#### ^15^N-labelled mSUMO2 plus XRCC4^FL^

Colour gradient range for mSUMO2 structure (PDB 2N1W) and sequence was based on intensity losses in the ^1^H–^15^N HSQC spectra of mSUMO2 after addition of 0.33 molar equivalents of XRCC4^FL^ as follows: from red (most affected by binding: <20% intensity after addition of 0.33 mSUMO2 equivalents) to blue (unaffected by 0.33 molar equivalents), with intermediate points as follows: 21–30% intensity at 0.33 equivalents, 31–40% intensity at 0.33 equivalents, 41–66% intensity at 0.33 equivalents.

#### ^15^N-labelled mSUMO2 plus PIAS2 peptide

Colour gradient range for mSUMO2 structure (PDB 2N1W) and sequence was based on intensity losses in the ^1^H–^15^N HSQC spectra of mSUMO2 after addition of 0.5 mSUMO2 equivalents of PIAS2 peptide (467-VDVIDLTIESS-477) as follows: from red (most affected by binding: <20% intensity after addition of 0.5 mSUMO2 equivalents of PIAS2 peptide) to blue (unaffected by 0.5 equivalents), with intermediate points as follows: 21–30% intensity, <31–40% intensity and 41–66% intensity.

#### ^15^N-labelled mSUMO1 plus XRCC4^1–164^

Colour gradient range for mSUMO1 structure (PDB 4WJQ) and sequence was based on chemical shift perturbations in the ^1^H–^15^N HSQC spectra of mSUMO1 after addition of 1 mSUMO1 equivalent of XRCC4^1–164^ as follows: from red (most affected by binding: CSP > 4× standard deviation) to blue (unaffected, <1 standard deviation), with intermediate points as follow, 2–4 standard deviations, 1–2 standard deviations. The standard deviation was 0.0012 ppm, although spectral resolution was 0.0078 ppm for ^1^H.

### Docking simulations

Docking simulations were performed using HADDOCK (15). NMR intensity losses were used to generate ambiguous interaction restraints. For the interaction between the head domain of XRCC4^1–164^ and SUMO2, the ‘active’ residues were 57, 59, 102, 103, 104, 105 and 106 for XRCC4 and 30, 31, 33, 35, 40, 41, 42, 50 and 51 for mSUMO2. The ‘passive’ residues were 56, 62, 65, 99, 101, 109 and 111 for XRCC4 and 29, 36, 38, 43, 68, 84, 86 for mSUMO2. The docked protein structures were based on PDB entries 1IK9 for XRCC4 (chain A) and 2D07 for SUMO2. In order to assess the reproducibility, the HADDOCK docking was repeated with one active residue omitted from each binding partner for each possible pair. The 63 docked structures generated, clustered into seven classes, and these seven clusters showed only two orientations of mSUMO2 relative to XRCC4. These two orientations were used to generate a model of 4×SUMO2 binding to XRCC4, adding two intervening copies of SUMO2 with no interactions, and placed arbitrarily except to ensure the continuity of the peptide chain. This model was then minimised and equilibrated by molecular dynamics using GROMACS (MD step used 5 ns in 2 fs steps using the AMBER99SB-ILDN forcefield and TIP3P water). For the interaction between the coiled-coil region of XRCC4 and SUMO2, the ‘active’ residues for XRCC4 were 164, 165, 166 and 167, with no ‘passive’ residues, and the same set of ‘active’ and ‘passive’ residues was used for SUMO2. For this complex, there were too few interacting residues to attempt the omission strategy. HADDOCK generated a single cluster for this complex. This complex and the most common (model 1) complex of the SIM56/SIM101 interaction were used to generate models of di- and tri-SUMO2 complexes, which span both binding sites. This was minimised and equilibrated using the same molecular dynamics strategy.

### Surface plasmon resonance (SPR)

Experiments were performed on a ProteOn XPR36 instrument (BioRad Laboratories) using a running buffer containing 100 mM NaCl, 10 mM HEPES pH 7.0 and 0.1% (v/v) Igepal. Recombinant XRCC4-6×His was immobilised on a GLC chip (BioRad Laboratories) in the vertical orientation. Chip channels were activated using a mixture of 25 mM *N*-ethyl-*N*′-(3-dimethylaminopropyl) carbodiimide (EDC) and 15 mM sulfo-*N*-hydroxysuccinimide (sulfo-NHS). Proteins were immobilised in 10 mM sodium acetate buffer, pH 4.5 (BioRad Laboratories). Remaining crosslinking sites were blocked by injection of 150 μl of 1 M ethanolamine–HCl (pH 8.5). Different SUMO or ubiquitin topologies were run in horizontal orientation at concentrations specified in each panel. Surface regeneration was accomplished with a pulse of 50 mM NaOH at 100 μl/min. All experiments were performed at 25°C. Regression curves were obtained via non-linear regression based on a single-site binding model (GraphPad Prism v6.0h).

### Biolayer interferometry (BLI)

BLI was performed using an Octet RED96 instrument (ForteBio). 50 μg of recombinant 6×His-4×SUMO2-Strep were biotinylated using EZ-link NHS-PEG4-Biotin (Thermo Fisher) following the manufacturer's protocol. Excess biotin was removed using Zeba desalting spin columns (Thermo Fisher). 1 μg of biotinylated 6×His-4×SUMO2-Strep was immobilised on streptavidin (SA) biosensors (ForteBio) until an approximately 1000 nm response was reached. The baseline was set by submerging 4×SUMO2-captured sensors in kinetics buffer (PBS+0.02% Tween-20, 0.1% BSA, 0.05% NaN_3_) in a 96-well plate, integrating an orbital shake function. The binding curves were obtained by dipping the sensors in 96-well plates containing the analytes diluted in kinetics buffer, or kinetics buffer only as reference. Finally, the sensors were dipped in fresh kinetics buffer for the dissociation step. Sensors were regenerated using 100 mM glycine pH 2.5 prior to reuse. Unloaded SA biosensors were used as controls and subtracted where unspecific binding was observed.

### Photo-induced crosslinking assays

Recombinant proteins with the unnatural amino acid *para*-l-benzoyl-phenylalanine (BpF) for crosslinking experiments were produced in *Escherichia coli* BL21 (DE3) Gold cells transformed with the plasmid pEVOL-pBpF, a gift from Peter Schultz (Addgene plasmid #31190; http://n2t.net/addgene:31190; RRID:Addgene_31190) (16) for the expression of the orthogonal BpFRS/BpFtRNA_CUA_ pair derived from *Methanococcus jannaschii*. Proteins were expressed in LB media after the addition of IPTG, 0.02% arabinose and 1 mM BpF. The crosslinking assay was carried out as previously described (17,18). Briefly, BpF-containing proteins (final concentration 10 μM for SUMO topologies, 20 μM for XRCC4^1–164^) were incubated with the respective binding partner (10 μM for SUMO, 20 μM for XRCC4^1–164^) for 15 min at 4°C in 0.2 ml thin-walled polypropylene PCR tubes (Greiner Bio One). The samples were irradiated for 1 h with long-wave UV light (λ=365 nm; Herolab UV-16 L, 8 W, 6 mm distance). For SDS-PAGE analysis, samples were taken before and after UV irradiation. For LC–MS/MS analysis the crosslink-bands were excised from the SDS-PAGE gels and digested with trypsin. Samples were analysed in an UltiMateTM 3000 RS LC nano system (Thermo Fisher Scientific GMbH) connected to a maXis II UHR-qTOF mass spectrometer with a nano-ESI source (CaptiveSpray with nanoBooster, Bruker Daltonik GmbH). To determine the crosslinked peptides the LC–MS/MS output files were analysed using the free software StavroX version 3.6.0.1 (http://www.stavrox.com) that generates all theoretical possible crosslinked peptides. The identified potential crosslinked peptides were scored by the comparison of their theoretical fragmentation to the measured LC–MS/MS spectrum and only scores above a hundred were considered real crosslinks.

### Cell culture

HEK293(T) cells were cultured in high-glucose Dulbecco's Modified Eagle Medium (DMEM, Sigma), supplemented with 10% (v/v) fetal bovine serum (FBS, Sigma), 2 mM l-glutamine, 100 U/ml penicillin, and 100 μg/ml streptomycin (Gibco) at 37°C in a humidified atmosphere at 5% CO_2_. Transfections were carried out using Fugene6 (Promega) according to the manufacturer's instructions. Ionizing radiation (IR) treatments were performed using a CellRad Faxitron instrument (Faxitron Bioptics, LLC).

### Construct design

The GST-diSUMO2 plasmid was generated by overlapping PCRs of SUMO2 lacking the final two residues fused to a truncated SUMO2 (corresponding to amino acids 11–95) with a di-glycine linker in between the two SUMO2 units and inserted into the BamHI/EcoRI sites of the pGEX-2T vector. XRCC4^1–180^ (cysteines 93, 128 and 130 mutated to alanines (C-to-A)) for NMR purposes was amplified from the pET-28a-XRCC4^1–164^ (C-to-A) vector with a reverse primer containing the sequence for residues 165–180 and inserted into the pET-28a vector linearised with NcoI/EcoRI, and validated by sequencing. XRCC4^1–164^ SIM101-5A (cysteines 128 and 130 mutated to alanines), SIM101-AENTA (cysteines 128 and 130 mutated to alanines), SIM56-5A (cysteines 93, 128 and 130 mutated to alanines), SIM56-AKKAA (cysteines 93, 128 and 130 mutated to alanines), SIM56/101-10A (cysteines 128 and 130 mutated to alanines) were generated by overlapping PCRs with mutagenic primers using the pET-28a-XRCC4^1–164^ as template. The XRCC4^1–164^ 2KE mutant was amplified from the full-length XRCC4 K65E K99E mutant (19), a kind gift from Murray Junop (Western University London, USA), and inserted into the pET-28a vector linearised with NcoI/EcoRI, and validated by sequencing. XLF was amplified from pGEX2TKP-XLF (20) and subcloned into the pHAT5 vector. XRCC4 truncated versions XRCC4^1–172^, XRCC^1–180^, XRCC4^1–213^ and XRCC4^1–270^ were amplified from pHAT5-XRCC4^FL^. XRCC4 pSIM8, pSIM33, pSIM123 and pSIM181, with the 5 amino acids of each of the pSIMs mutated to alanines, were generated by overlapping PCRs with mutagenic primers using pHAT5-XRCC4^FL^ as template and inserted into the NcoI/BamHI sites of the pHAT5 expression vector. STMN1 was amplified from the Gateway entry clone pDONR223-STMN1, a kind gift from Alfred Vertegaal, and inserted into the NcoI/BamHI sites of the pHAT5 vector. STMN1 SIM43-5A (43-KDLSL-47 to alanines) was generated by overlapping PCRs with mutagenic primers using pDONR223-STMN1 as template and inserted into the NcoI/BamHI sites of the pHAT5 vector. The following plasmids were used for mammalian transfections: pEGFP-C1-FLAG-XRCC4, pEGFP-C1 (Clontech) containing FLAG-XRCC4 (denoted GFP-XRCC4, unless stated otherwise), has been described previously (21). XRCC4 SIM101-5A, XRCC4 SIM163-5A and XRCC4 SIM101/163-10A, which have 101-LKDVS-105, 163-EKCVS-167 or both regions mutated to alanines, respectively, were generated by overlapping PCRs with mutagenic primers using pEGFP-C1-FLAG-XRCC4 as template and inserted into the XhoI/EcoRI sites of pEGFP-C1-FLAG. pEGFP-C1-FLAG was generated by removing XRCC4 from pEGFP-C1-FLAG-XRCC4 using BamHI. Mammalian expression plasmids containing FLAG-XRCC4 WT, SIM101-5A, SIM163-5A and SIM101/163-10A were generated by amplifying FLAG-XRCC4 WT or the FLAG-XRCC4 SIM101–5A, SIM163-5A and SIM101/163-10A from the pEGFP-C1-FLAG-XRCC4 WT or SIM mutant plasmids, respectively, and inserted into the AflII/EcoRI sites of the pCDNA3.1 vector.

### Streptavidin pulldowns

6×His-4×SUMO2-Strep was biotinylated as described above. 30 μg of biotinylated protein were mixed with 60 μl of streptavidin agarose resin (Thermo Fisher) and rotated (end-over-end) for 30 min at RT. The 4×SUMO2-bound beads were then centrifuged and washed. 30 μg of 6×His-XRCC4 diluted in 500 μl of PBS containing 1% Igepal were added to the 4×SUMO2-captured beads and rotated (end-over-end) for 1 h at RT. Subsequently, the beads were washed 5 times in PBS containing 1% Igepal, and resuspended in 2× SDS Laemmli buffer (120 mM Tris–HCl pH 6.8, 4% SDS, 20% glycerol, 0.02% bromophenol blue and 2.5% β-mercaptoethanol). Proteins were visualised with InstantBlue stain (Expedeon).

### GFP immunoprecipitations

HEK293T cells transfected with the desired expression construct were washed with ice-cold PBS and scraped into lysis buffer (50 mM Tris–HCl pH 7.5, 150 mM NaCl, 10% glycerol, 2 mM MgCl_2_, 10 mM *N*-ethylmaleimide) with 1× Complete EDTA-free protease inhibitors (Roche) and 6 μl benzonase (Millipore) and rotated at RT for 15 min. Subsequently, the lysates were centrifuged at 16 000 g for 60 min and the supernatant bound to 25 μl of GFP-Trap magnetic beads (Chromotek) for 1 h with end-over-end rotation at 4°C. Protein-bound beads were then washed 5 times with lysis buffer and resuspended in 2× SDS Laemmli buffer (120 mM Tris–HCl pH 6.8, 4% SDS, 20% glycerol, 0.02% bromophenol blue and 2.5% β-mercatoethanol). For competition experiments the indicated amount of recombinant 6×His-4×SUMO2-Strep was added to the lysates prior to the incubation with the beads. 4% of input lysate were loaded unless stated otherwise.

### GST precipitations

Pulldowns with cellular extracts were carried out as described for GFP-immunoprecipitations using 1–3 μg of GST-fused proteins bound to glutathione magnetic beads (Promega). For immunoprecipitations of recombinant proteins, 1–3 μg of GST-fused proteins bound to glutathione magnetic beads were resuspended in 600 μl of binding buffer (50 mM Tris–HCl pH 7.5, 150 mM NaCl, 10% glycerol, 2 mM MgCl_2_, 10 mM *N*-ethylmaleimide) containing 2 μg or equimolar concentrations of His-tagged recombinant proteins and incubated for 1 h with end-over-end rotation at 4°C. Protein-bound beads were then washed 5 times with lysis buffer and resuspended in 2× SDS Laemmli buffer (120 mM Tris–HCl pH 6.8, 4% SDS, 20% glycerol, 0.02% bromophenol blue and 2.5% β-mercatoethanol). 4% of input lysate were loaded unless stated otherwise. Proteins were visualised with InstantBlue stain (Expedeon) or by immunoblotting.

### Aggregation onset temperature (*T*_agg_) determination

An Uncle (Unchanged Labs) platform was used to measure intensities of static light scattering (SLS) at 260 nm over a temperature ramp ranging from 20°C to 95°C. Light scattering of a 260 nm laser was monitored from samples at a concentration of 0.5 mg/ml, which were forced to aggregate by raising the temperature at 1°C intervals. The aggregation onset temperatures (*T*_agg_’s) were calculated using Uncle software (version 2.0). SLS (260 nm) values were set to zero at the start of the temperature ramp.

### Microscale thermophoresis (MST)

Recombinant XRCC4 was fluorescently labeled using the Monolith protein labeling kit RED-NHS (amine reactive) dye (NanoTemper Technologies), following the manufacturer’s guidelines. Labeled XRCC4 was diluted in the appropriate buffer (PBS, 0.05% Tween-20) to a final concentration of 5 nM. Non-labeled recombinant 4×SUMO2 protein was serially diluted 1:1 in the same buffer, using 16 doubling dilutions from a starting concentration of 500 nM. Equal volumes of the labeled protein were mixed with each dilution, before sample loading into Monolith Pico premium capillaries (NanoTemper Technologies). Thermophoresis was performed at RT, using a Monolith PICO instrument controlled with NT Control software version 1.0.1 with 5, 30, 5 s laser off, on, off times, respectively, and 5% LED power and medium IR-laser (MST) power. Experiments were carried out in triplicate and analysed using the NT Affinity Analysis software version 2.0.2 and applying the *K*_D_ model of fit. Data points displaying irregularities or where the MST/TRIC traces showed bleaching and/or artefacts from aggregation were defined as outliers, as previously described (22).

### EJ7-GFP reporter system

The HEK293 EJ7-GFP XRCC4 knock-out (KO) cell line (23) was used to generate the XRCC4 KO, XLF KO double KO cell line, using an sgRNA/Cas9 plasmid targeting XLF, as described previously (23). The expression plasmids for targeting DSBs in the EJ7 reporter (px330-7a and px330-7b), pCAGGS-3×Flag-XLF-WT, pCAGGS-3×Flag-XLF-L115D, empty vector control for XLF (pCAGGS-BSKX), and GFP expression vector (pCAGGS-NZEGFP), were previously described (23). Cells were seeded on a 24-well dish at 0.5 × 10^5^ cells per well, and transfected the following day as described (23), using 1.8 μl of Lipofectamine 2000 (Thermo Fisher) in 0.5 ml using 200 ng pX330-7a, 200 ng px330-7b, 50 ng XRCC4 plasmid (FLAG-XRCC4-WT, FLAG-XRCC4-SIM101-5A, FLAG-XRCC4-SIM163-5A, FLAG-XRCC4-SIM101/163-10A (double, D), as described in ‘Construct design’ section above) or empty vector (EV), 50 ng XLF expression plasmid or EV. For the transfection efficiency control, 200 ng pCAGGS-NZEGFP and 200 ng pCAGGS-BSKX were used in place of the px330 plasmids. For immunoblot analysis, transfections were the same final concentrations, with pCAGGS-BSKX replacing the px330 plasmids. Three days after transfection, cells were analysed for frequency of GFP+ using an ACEA Quanteon, and repair frequencies were normalised to parallel transfection efficiency controls, as described (23).

### Immunoblotting

The following primary antibodies were used: anti-XRCC4 (sc-271087, Santa Cruz, 1:100–1000), anti-mCherry (632543, Takara, 1:1000), anti-SUMO2/3 (ab3742, Abcam, 1:1000), anti-GFP (11814460001, Roche, 1:1000), anti-XLF (ab33499, Abcam, 1:500 or Bethyl A300-730A, 1:1000), anti-LIG4 (ab193353, Abcam, 1:1000), anti-DNA-PKcs (sc-5282, Santa Cruz, 1:100), anti-GST (27457701V, G&E, 1:1000), anti-6×His tag (MA1-21315, Invitrogen, 1:1000), anti-tubulin (Sigma T9026, 1:1000), anti-FLAG (20543–1-AP, Proteintech, 1:2000–4000), anti-FLAG-HRP (Sigma A8592, 1:1000), and anti-MLH1 (Abcam ab92312, 1:1000). The following HRP-conjugated secondary antibodies were used: anti-mouse (P0260, Dako, 1:10 000), anti-rabbit (31462, Invitrogen, 1:10 000) and anti-goat (P0449, Dako, 1:10 000). Proteins separated by SDS-PAGE were detected following the manufacturer's guidelines (GE Healthcare ECL Western Blotting detection system) and the images collected using a Chemidoc imaging system (BioRad Laboratories).

### Recombinant proteins and peptides

Proteins were expressed in *E. coli* BL21-CodonPlus (DE3)-RIL (Stratagene) in Luria-Bertani (LB) medium unless specified otherwise. 6×His-tagged full-length XRCC4 (24), XRCC4^1–213^ (C-to-A: cysteines mutated to alanines) (25), XRCC4^1–164^ (C-to-A: cysteines mutated to alanines) (26) expression plasmids were a generous gift from Tom Blundell (University of Cambridge, UK). All XRCC4 recombinant constructs were purified as described previously (25). The 6×His-4×SUMO2-Strep expression plasmid was a kind gift from Cynthia Wolberger (Johns Hopkins University, USA) (27). 6×His-4×SUMO2-Strep, a linear fusion of an N-terminal full-length SUMO2 fused to truncated SUMO2 (residues 11–92) linked via K11 for the second, third and fourth, was expressed and purified as described previously (27). Bacterial expression plasmids pAS2974 (encoding GST-SUMO1) (28), pAS2976 (encoding GST-SUMO2) (28) and pAS4179 (encoding GST-4×SUMO2, a linear SUMO2 chain consisting of four truncated SUMO2 (residues 12–93) moieties fused to an N-terminal GST tag) (29) were kindly provided by Andrew Sharrocks (University of Manchester, UK). GST-SUMO1, GST-SUMO2 and GST-diSUMO2 were purified using glutathione sepharose 4B beads (GE Healthcare) according to the manufacturer's guidelines, followed by overnight on-bead thrombin digestion (Sigma) and size exclusion chromatography using a Hiload 16/600 Superdex 75 prep grade column (GE Healthcare). GST-4×SUMO2 was purified using MagneGST Glutathione particles (Promega) as previously described (30). XLF-6×His was purified using a HP His-trap column followed by a HiTrap Q HP column (both GE Healthcare). mSUMO1 (#UL-712), mSUMO2 (#UL-752), and SUMO2/3 chains enzymatically linked via their internal K11 residues—diSUMO2 (#ULC-200), polySUMO2 (#ULC-210) and polySUMO3 (#ULC-220)—were purchased from Boston Biochem. Recombinant 6×His-XLF (NBP2-23291), used for SPR experiments, and GST-TCEAL6 (H00158931-P01) were purchased from Novus Biologicals. PAK3-6×His (11532-H08B), 6×His-WARS (14827-H07B), 6×His-DARS (14278-H07E) and 6×His-STMN1 (15440-H07E) were purchased from Sino Biological. 6×His-SSB (ab84477) and 6×His-RBBP5 (ab268918) were purchased from Abcam. The peptides used in this study were purchased from Genosphere Biotech. All dialyses were carried out using Spectra/Por 3 kDa MWCO dialysis membranes (Spectrum labs) at 4°C. Proteins were concentrated using Vivaspin PES concentrators (Generon). Protein purity was assessed by protein staining of SDS-PAGE gels.

^15^N-labelled proteins for NMR experiments were expressed in minimal medium supplemented with ^15^NH_4_Cl. ^15^N-^13^C-labelled recombinant XRCC4^1–180^ for NMR assignment was expressed in minimal medium supplemented with ^15^NH_4_Cl and ^13^C-glucose. ^15^N–^13^C–^2^H-labelled recombinant XRCC4^1–164^ for NMR assignment was expressed in minimal medium supplemented with ^15^NH_4_Cl and ^13^C-glucose and using D_2_O instead of H_2_O for 8 h at 37°C after IPTG induction. Labelled proteins were purified as described above.

## RESULTS

### Proteome microarray screening retrieves polySUMO2 receptors with diverse gene ontologies

**Figure 1.**
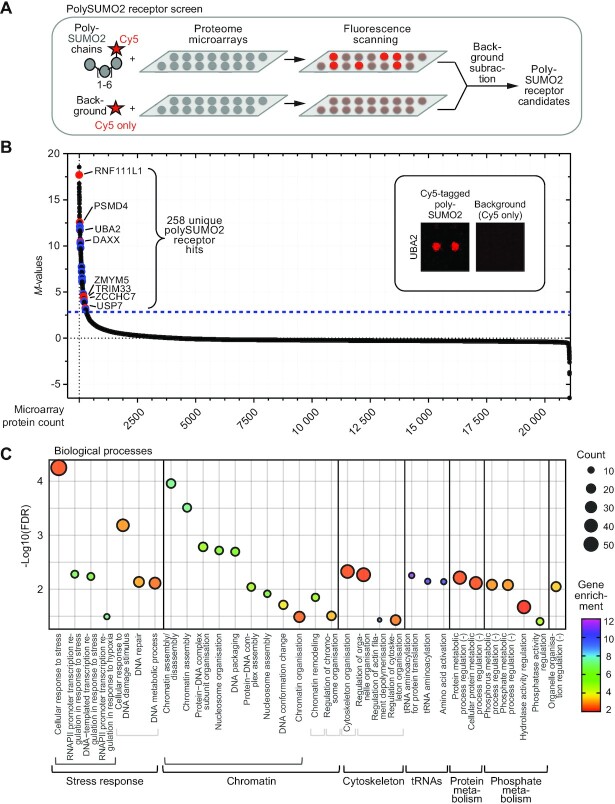
Proteome microarray screen identifies novel polySUMO2 receptors. (**A**) Human proteome microarray screening pipeline. (**B**) Screening results highlighting known/described SUMO2 receptors (red) and ones validated in this study (blue). Dashed blue line indicates the statistical threshold for polySUMO2 receptor candidates. (**C**) Gene ontology enrichment for biological processes enriched >2-fold in SUMO receptor candidate list, using Panther Classification System. (–) denotes negative regulation. FDR: false discovery rate; RNAPII: RNA polymerase II.

SUMO receptors have mainly been identified with yeast-two-hybrid systems or affinity purification from whole cell extracts using SUMO topologies as baits combined with mass spectrometry (5,29,31,32,33,34,35). These techniques are limited by their propensity to identify indirect SUMO binders in addition to direct, binary receptors. Additionally, mass spectrometry-based approaches are restricted by the cell-tissue-specific proteomes used as starting materials and biased towards abundant proteins. A recent photo-crosslinking approach targeted binary receptors (direct SUMO binders as opposed to proteins indirectly associating with SUMO via SUMO-independent protein interactions with the direct binders), but was limited to the identification of receptors interacting with a specific region on SUMO2 (18). To overcome these restrictions, we systematically screened the human proteome for polySUMO2 receptors using microarrays containing duplicate protein spots for >21 000 proteins representing ∼15 000 unique full-length human genes with one or more isoforms. To this end, we incubated the arrays with enzymatically linked polySUMO2 chains fluorescently labelled with Cy5, followed by fluorescence scanning and background subtraction using soluble Cy5 as a reference (Figure 1A, Supplementary Table S1). The screen identified a total of 258 unique binary polySUMO2 receptor candidates (Figure 1B; Supplementary Table S2), featuring known SUMO2 receptors and numerous proteins with no previous SUMO-binding functions assigned to them. As expected, known/predicted SUMO receptors harboured components of the SUMO conjugation cascade, in addition to downstream receptors with no known SUMOylation roles (Figure 1B). Moreover, SUMO receptors with known preferences for SUMO1 binding, e.g. RGS17, DPP9 and PARK2, did not score as hits, suggesting that our approach was able to distinguish between different SUMO paralogues.

SUMOylation is known for its importance in regulating processes inside the nucleus and in response to stress (1,9). Consistent with this notion, gene ontology analysis of the polySUMO2 hits resulted in a significant enrichment of biological processes linked to stress-induced transcription and DNA repair, in particular in response to hypoxia and DNA damage (Figure 1C). These findings underpin the validity of our approach in identifying receptors in pathways associated with known SUMO functions, and in contexts where the formation of polySUMO chains is triggered, an achievement previously unattained with proteome microarrays (36). Other significantly enriched ontologies included chromatin-associated processes such as remodelling and nucleosome assembly/disassembly, also linked to SUMO function (37) (Figure 1C). Significant enrichment was also observed for processes less established for regulation by SUMOylation, such as cytoplasmic cytoskeletal organisation, consistent with SUMOylation emerging as an important regulatory layer in that arena (38), and tRNA aminoacylation. Finally, our analyses connected SUMO processes to phosphorylation (Figure 1C), with cross-talk between different PTMs representing an emerging and exciting theme.

### Validation of polySUMO2 receptors with diverse functions and binding characteristics

**Figure 2.**
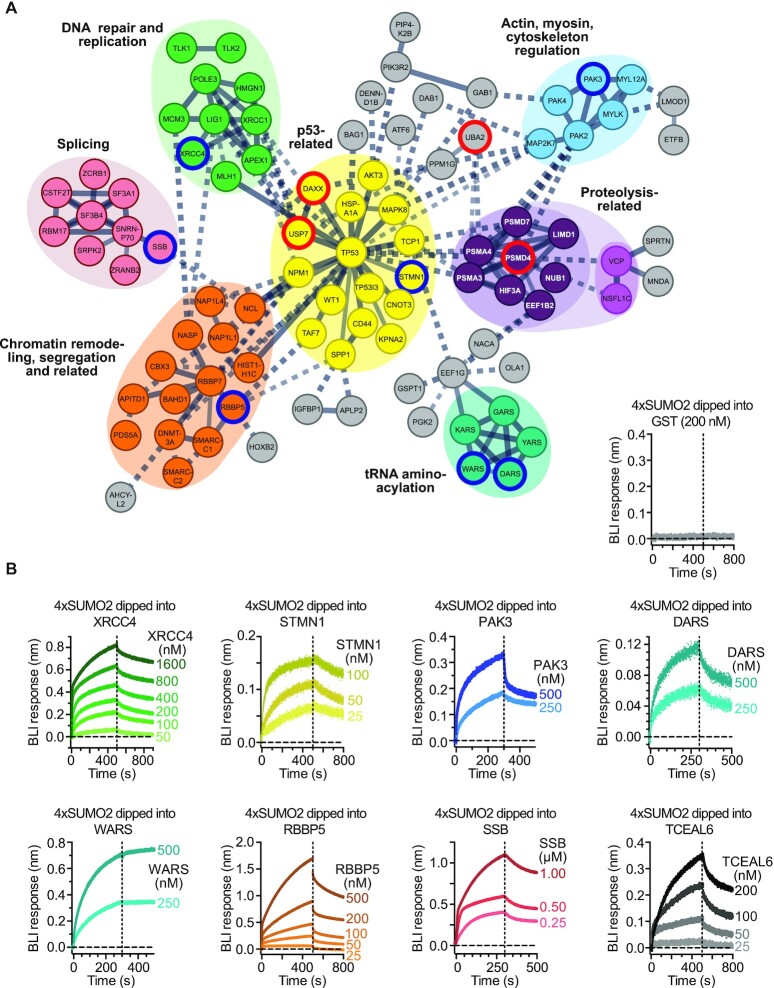
Network analysis and validation of polySUMO2 receptor candidates. (**A**) Clusters of polySUMO2 receptor candidates identified by STRING network analysis (75). Red and blue strokes highlight known SUMO receptors and receptors validated in this study, respectively. (**B**) Validation of polySUMO2 chain receptor candidates across different gene clusters or with no consensus SUMO-interacting motifs (SIMs) present in their sequence (TCEAL6) using biolayer interferometry (BLI). Colouring according to corresponding gene clusters in (A), or in black/grey for TCEAL6. Vertical dashed lines separate association and dissociation phases.

With a range of biological processes significantly enriched amongst the hits, we tested if the candidates were functionally interconnected. STRING network analysis revealed a significant enrichment of protein:protein interactions (enrichment *P* value=1.51×10^–12^), and several gene clusters including a central hub of p53-associated processes (Figure 2A). Some, but not all, of these clusters were linked to SUMO-associated functions (known SUMO receptors highlighted with red borders in Figure 2A). For example, the coordinated functions of DAXX and USP7 to regulate p53 function (39) connected them to the central cluster, and PSMD4 linked to other proteasomal components not previously associated with SUMO binding. In this regard, both VCP (aka p97) and one of its interaction partners, NSFL1C, came up as hits. A different interaction partner of VCP, UFD1, functions as a SUMO receptor in yeast to help recruit the VCP complex to its targets (40), raising the possibility that the VCP complex takes on similar roles in humans. Other gene clusters formed by SUMO receptor candidates centred on nuclear functions including DNA repair, chromatin regulation and pre-mRNA splicing, consistent with enrichment of these pathways in our gene ontology analysis (Figure 1C). Interestingly, several serine/threonine kinases (p21-activating kinases; PAKs) formed part of a gene cluster linking cytoplasmic cytoskeleton functions with nuclear signalling. Moreover, five aminoacyl tRNA synthetases formed a separate cluster, representative of the strong enrichment of tRNA-associated processes in the identified gene ontologies (Figure 1C). While none of these proteins had previous SUMO receptor functions assigned to them, tRNA transcription is regulated by SUMOylation in response to stress in yeast (41). This raises the possibility that SUMOylation and SUMO receptor interactions, in tRNA-mediated protein translation, could help cells respond to stress, thereby offering a starting point to assess such functions mechanistically in the future.

To assess the validity of the identified receptor candidates, we performed SUMO-binding assays on a range of candidates using biolayer interferometry (BLI). Using tetraSUMO2 chains, we validated eight candidates distributed across a range of gene clusters and *M*-values (Figure 2B; Supplementary Table S1). These included XRCC4, a DNA repair protein; STMN1, a cytosolic protein regulated by p53 and recently hypothesised to function via SUMO binding (42); PAK3, a serine/threonine protein kinase; DARS and WARS, two aminoacyl-tRNA synthetases; RBBP5, a transcriptional regulator associated with histone methyltransferase complexes; SBB, a protein important for RNA metabolism (Figure 2A, genes with blue frames); and TCEAL6, a transcriptional elongation factor for which no putative SIMs were retrieved using the SIM prediction servers JASSA and GPS-SUMO (4,43). The validated receptors displayed distinct association and dissociation profiles, with some associating more stably with 4×SUMO2 than others (Figure 2B, compare e.g. WARS with TCEAL6). The presence of at least one validated SUMO2 receptor in every identified gene cluster emphasises the validity of our screening approach. Moreover, the findings demonstrate the ability of our setup to identify receptors with distinct binding characteristics, featuring diverse biological functions linked to established and hitherto undiscovered aspects of SUMO binding and functionality.

### XRCC4 preferentially binds polySUMO2/3 chains

**Figure 3.**
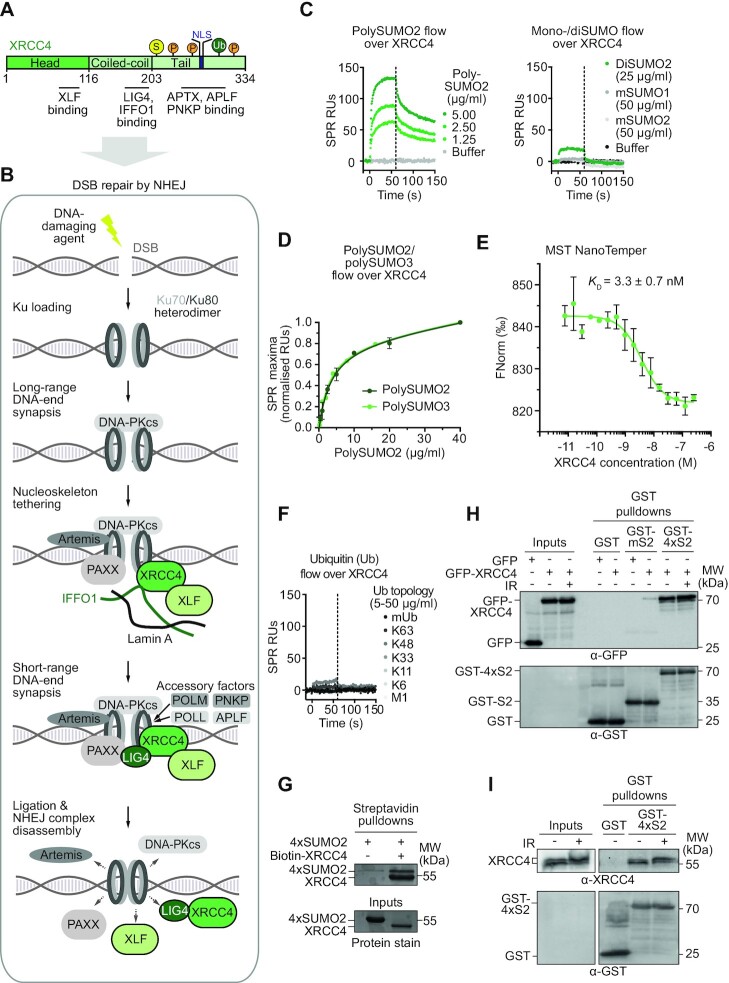
XRCC4 preferentially binds to polySUMO2 chains. (**A**) Protein schematic of XRCC4 highlighting key structural and functional features. S: SUMOylation; P: phosphorylation; Ub: ubiquitylation; NLS: nuclear localisation signal. (**B**) Schematic of DNA double-strand break (DSB) repair by non-homologous end-joining (NHEJ) highlighting core NHEJ factors and selected accessory factors. Note that the linearity of the indicated steps and simultaneous interactions of proteins in several of the complexes remain largely unknown. (**C**) Preferential binding of XRCC4 to polySUMO2 chains (left) over SUMO monomers (mSUMO1, mSUMO2) and dimers (diSUMO2; right) by surface plasmon resonance (SPR). Vertical dashed lines separate association and dissociation phases. (**D**) Equilibrium analysis of SPR response unit (RU) maxima of polySUMO2/3 binding to immobilised wildtype, full-length XRCC4. (**E**) NanoTemper microscale thermophoresis (MST) titration using XRCC4 as ligand and 4×SUMO2 as binding partner. *K*_D_: dissociation constant. Error bars represent means±standard deviations of *n*=3 replicates. (**F**) SPR sensorgram showing no or minor detectable binding of XRCC4 to ubiquitin monomers (mUb) and tetra-ubiquitin chains, as indicated. Vertical dashed lines separate association and dissociation phases. (**G**) Recombinant protein pulldowns showing XRCC4 interaction with 4×SUMO2 (6×His-4×SUMO2-Strep) in solution *in vitro*. (**H**) GST-pulldowns of SUMO2 monomers (mS2) and 4×SUMO2 pulldowns co-precipitating GFP-XRCC4, ectopically expressed in HEK293T XRCC4 KO cells in the absence or presence of DNA damage induced by ionizing radiation (IR; 15 Gy, ∼15 min). (**I**) GST-4×SUMO2 pulldown co-precipitates endogenous XRCC4 from HEK293T nuclear extracts in the absence or presence of IR (15 Gy, ∼15 min). Pulldowns were repeated twice with similar results. Inputs were 4% of the total.

To further define the SUMO-binding characteristics of one of the receptors arising from our screen, we selected the core NHEJ factor XRCC4 for follow-on studies (*M*-values ∼6; Figure 1B and Supplementary Table S1). SUMOylation is crucial for efficient NHEJ (44,45,46). Despite this importance, knowledge of SUMO receptor roles for core NHEJ factors has long been lacking and only recently started to come to light during the course of this study. XRCC4 is a 38 kDa protein mainly existing as a homodimer in cells and featuring a number of functionally and structurally distinct domains (25). Its N-terminal head domain is important for interaction with another NHEJ core factor, XLF, followed by a coiled-coil domain that mediates binding with the NHEJ ligating enzyme LIG4 or the nucleoskeleton protein IFFO1 (25,47,48). A flexible C-terminal tail contains various phosphorylation sites important for mediating interactions with further NHEJ-associated DNA repair factors (49) and for removal of XRCC4 from chromatin (50) (Figure 3A). NHEJ is initiated in response to DSBs with Ku, a heterodimer formed by Ku70 and Ku80, recognising and binding broken DNA ends. Amongst several functions, DNA-bound Ku acts as a recruitment hub for other NHEJ factors e.g. the DNA damage response kinase DNA-PKcs, which together with Ku forms the holoenzyme DNA-PK (Figure 3B). DNA-PK itself can recruit other factors important for preparing the broken DNA ends for ligation (51). XRCC4 is key for facilitating distinct aspects of NHEJ. By interacting with XLF, XRCC4 is implicated in tethering DNA ends and thus, in promoting synapsis and subsequent DNA-end ligation, although the importance of this seems to depend on cellular context (52,53,54,55,56,57). Moreover, interaction with XRCC4 stabilises LIG4 and promotes its enzymatic activity, thereby facilitating the final NHEJ ligation step (Figure 3B) (58).

Given that different topologies of SUMO are associated with distinct functions (59), we first assessed if XRCC4 displayed preferential binding to different SUMO topologies. Surface plasmon resonance (SPR) assays revealed preferential binding of XRCC4 to enzymatically linked polySUMO2 chains (Figure 3C, left) over SUMO1/2 monomers (mSUMO1/2) and SUMO2 dimers (diSUMO2; Figure 3C, right). In agreement with the high sequence identity between SUMO2 and SUMO3, XRCC4 bound polySUMO2 and polySUMO3 chains in a similar manner (Figure 3D). It is noteworthy that the heterogeneous nature of these chains (3–8 SUMO moieties at different quantities) precluded calculation of their *K*_D_ dissociation constants in molarity. Using 4×SUMO2 chains in NanoTemper microscale thermophoresis (MST) experiments, we retrieved a *K*_D_ of 3.2±0.7 nM (Figure 3E), which puts this interaction amongst the strongest known SUMO:receptor interactions (60). In this regard, we note that the strength of the XRCC4:4×SUMO2 interaction is specific for 4×SUMO2, with much weaker affinities applying to SUMO2 monomers (see NMR titrations below). These findings could be explained by local concentration effects or by the existence of multiple SIMs on XRCC4 that bind to distinct SUMO moieties of the same chain with increased avidity.

Since SUMO belongs to the ubiquitin/UBL family, we next investigated if XRCC4 showed a preference for binding to SUMO over ubiquitin topologies. We detected no/minor interactions of XRCC4 to a wide range of ubiquitin topologies (Figure 3F). Next, we performed tetraSUMO2 chain (4×SUMO2) co-precipitations with recombinant XRCC4, demonstrating that the two proteins can also interact in solution (Figure 3G). Additionally, XRCC4 bound to SUMO2 in cells; similar to the binding characteristics we established *in vitro*, ectopically expressed XRCC4 preferentially co-precipitated from cellular extracts with polySUMO2 chains over SUMO2 monomers (mSUMO2), irrespective of the presence or absence of ionizing radiation (IR)-induced DNA damage (Figure 3H). The interaction was also detectable with endogenous XRCC4 (Figure 3I). Overall, these findings demonstrate that XRCC4 can preferentially bind polySUMO2 chains *in vitro* and in cells, and in the absence or presence of DNA damage.

### XRCC4 lacks functional consensus SIMs

To characterise if and how the positioning of SUMO-binding regions on XRCC4 related to the structure and function of XRCC4’s known domains (Figure 3A), we next investigated if XRCC4 contained conventional SIM sequences, which feature a core of 4–5 hydrophobic residues that can be intersected or framed by negatively charged residues on one or both sides (Supplementary Figure S1a) (5,6,33). Indeed, JASSA and GPS-SUMO predicted five putative SIMs (pSIMs) in XRCC4: three located in its head domain (pSIM8, pSIM33 and pSIM123), one in its coiled-coil region (pSIM181), and one in the C-terminal part of the protein (pSIM257; Supplementary Figure S1b). However, the crystal structures of XRCC4 (25,61,62) suggested that pSIM8, pSIM33, and pSIM123 are important for the structural integrity of the XRCC4 head domain by forming extensive interactions with nearby XRCC4 residues. Moreover, pSIM33 is almost completely buried inside the XRCC4 head domain, rendering this motif inaccessible to surface interactions (61) (Supplementary Figure S1b), as also supported by hydrogen exchange experiments, showing that pSIM33 is inaccessible to solvent, and stably so for several days (14). Consistent with these realisations, mutation of pSIM8, pSIM33, or pSIM123 residues to alanines abolished XRCC4 interactions with polySUMO2/3 (Supplementary Figure S1c), in agreement with recently published work on pSIM33 (35). However, interaction with XLF (Supplementary Figure S1d), which binds to a distinct region on XRCC4’s head domain (26,52,63,64) (compare Supplementary Figure S1b to Figure 3A), was also abrogated, suggesting that these mutations seriously affect the structural stability of the N-terminal domain. In line with this notion, the pSIM mutants started aggregating at markedly lower temperatures than the WT in response to thermal unfolding (>20°C lower for pSIM8; >30°C lower for pSIM33 and pSIM123), confirming the strong destabilising impact of the mutations on the XRCC4 fold (Supplementary Figure S1e). The stronger effects of pSIM33 and pSIM123 over pSIM8 are consistent with their more enhanced disruption of SUMO2 and XLF binding (Supplementary Figure S1c,d). By contrast, mutation of pSIM181 residues to alanines neither affected polySUMO2/3 nor XLF binding of XRCC4 (Supplementary Figure S1c,d). In addition, deletion of XRCC4’s C-terminal tail containing pSIM257 did not markedly affect polySUMO2 binding of XRCC4 (Supplementary Figure S1f). We conclude that XRCC4 most likely interacts with SUMO2/3 via one or multiple non-conventional, hitherto unidentified binding module(s).

### Carbene footprinting determines distinct SUMO2-binding regions on XRCC4

**Figure 4.**
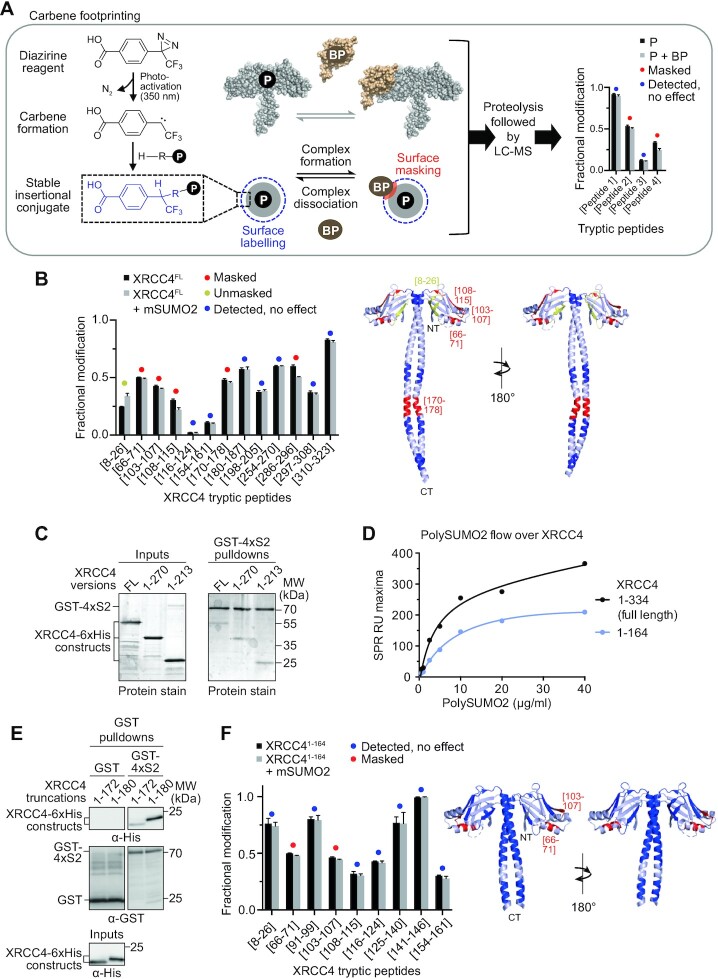
Carbene footprinting identifies distinct SUMO2-binding regions on XRCC4. (**A**) Experimental pipeline for carbene footprinting of the interaction between a protein (P)-of-interest and its binding partner (BP) using aryldiazirine as labelling probe. Fractional labelling, individually and in the presence of the BP, followed by proteolysis combined with liquid chromatography-mass spectrometry (LC–MS), allows comparative quantification of the labelling levels of the digested peptides. (**B**) Fractional modification by aryldiazirine of full-length XRCC4 (XRCC4^FL^) in the presence (grey bars) or absence (black bars) of SUMO2 monomers (mSUMO2). Significant peptide masking and unmasking differences (Student′s *t*-test, *P*<0.05) are highlighted in red or yellow, respectively, with unchanged peptide probing indicated in blue. The same colour code is applied to the structure of XRCC4^1–213^ (PDB 1IK9) (25) on the right, with undetected regions indicated in light blue. Error bars represent ±standard deviations based on *n*=3 independent biological replicates. (**C**) GST-4×SUMO2 pulldowns uncover comparable levels of recombinant XRCC4^FL^, XRCC4^1–270^ and XRCC4^1–213^ in the precipitated fractions. (**D**) Equilibrium analysis of SPR response unit (RU) maxima of polySUMO2 binding to immobilised XRCC4^FL^ (black), and XRCC4^1–164^ (blue) by surface plasmon resonance (SPR). (**E**) GST-4×SUMO2 pulldowns reveal higher levels of recombinant XRCC4^1–180^ in the precipitated fractions compared to XRCC4^1–172^. (**F**) Fractional modification by aryldiazirine of XRCC4^1–164^ in the presence (grey bars) or absence (black bars) of mSUMO2. Significant peptide masking and unmasking differences (Student′s *t*-test, *P*<0.05) are highlighted in red or yellow, respectively, with unchanged peptide probing indicated in blue. The same colour code is applied to the structure of XRCC4^1–164^ (PDB 1IK9) (25) on the right, with undetected regions indicated in light blue. Error bars represent ±standard deviations based on *n*=3 independent biological replicates. Pulldowns were repeated twice with similar results. CT: C-terminus; NT: N-terminus; RU: response unit; S2: SUMO2.

In light of the absence of conventional SIMs in XRCC4, we employed a recently developed structural mass spectrometry approach, known as carbene footprinting (12,13,65), to map SUMO2 interaction regions along XRCC4 in an unbiased manner. The carbene footprinting methodology utilises covalent labelling of surface-exposed protein residues with a highly reactive carbene species, formed by photolysis of the corresponding diazirine. Labelling of the protein-of-interest both individually and with a binding partner, followed by proteolytic digestion and LC–MS analysis, allows differential labelling of the resulting peptides to be monitored. Reduced peptide labelling in the presence of a binding partner, indicates the residues of the peptide as potential binding sites due to surface masking. In addition, unmasking of peptide labelling can occur, and both masking and unmasking can further indicate interaction-induced conformational changes leading to a change in exposure of residues to solvent, and therefore labelling (12,13,65) (Figure 4A).

Carbene footprinting of XRCC4 in the presence of mSUMO2 revealed multiple XRCC4 tryptic peptides as potential SUMO2-interacting sites. Significant masking was detected in XRCC4’s head domain for three peptides between residues 66–115, in the coiled-coil region (170–178 peptide; the preceding peptide was not detected in our analysis), and in the C-terminal tail in/around the 286–296 region. Significant unmasking occurred in the 8–26 region, an area on the head domain spatially proximal to the N-terminal part of the coiled-coil (Figure 4B, Supplementary Table S3). XRCC4 truncations lacking up to 121 amino acid residues of the C-terminus were precipitated by GST-4×SUMO2 with similar levels compared to full-length XRCC4 (Figure 4C), in agreement with the comparable polySUMO2 profiles we measured in SPR equilibrium analyses (Supplementary Figure S1f). These findings suggest that the masking of the 286–296 region was due to structural rearrangements of XRCC4’s C-terminus rather than its direct involvement in SUMO binding. A 1–164 XRCC4 truncation (XRCC4^1–164^) retained substantial polySUMO2 binding, consistent with XRCC4’s head domain contributing to SUMO binding (Figure 4D). In addition to the head domain, increased precipitation of XRCC4^1–180^ by GST-4×SUMO2 compared to XRCC4^1–172^ confirmed the presence of residues important for SUMO binding in the coiled-coil (Figure 4E). Taken together, these findings point towards SUMO-interacting regions on XRCC4 in its head and coiled-coil domains, with potential allosteric changes occurring at/around positions 8–26 and in the flexible C-terminus (286–296 positions). To increase the resolution of the carbene footprinting approach in the head domain, we repeated the analyses with XRCC4^1–164^, which retrieved an extended set of labelled peptides (Figure 4F, Supplementary Table S3). Overall, the experiment consolidated the effects we observed with full-length XRCC4, and strengthened our conclusion of there being at least two potential distinct SUMO-binding regions on the head domain localised to/around residues 66–71 and 103–107.

### Conserved non-conventional and paralogue-selective SUMO-binding module on XRCC4 head domain

**Figure 5.**
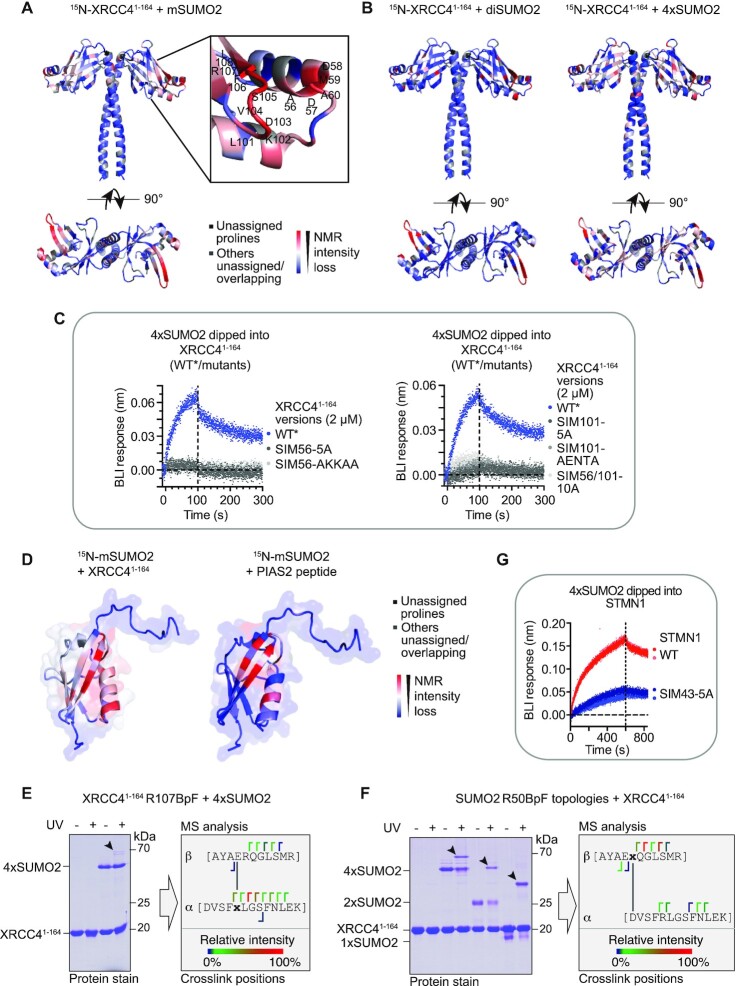
Unconventional SUMO2-specific binding of XRCC4 head domain. (**A**) Intensity losses in the ^1^H–^15^N BEST-TROSY spectra of XRCC4^1–164^ (PDB 1IK9) after addition of increasing concentrations of SUMO2 monomers (mSUMO2). Colour gradient: red (residues most affected by binding) to blue (residues unaffected by binding). Unassigned prolines displayed in black, other unassigned/overlapping residues in grey. Detailed view of key XRCC4^1–164^ residues affected is shown on the right. (**B**) XRCC4^1–164^ residues implicated in SUMO2 dimer (diSUMO2; left) and SUMO2 tetramer (4×SUMO2; right) binding, as indicated by intensity losses in the ^1^H–^15^N BEST-TROSY spectra of XRCC4^1–164^ after addition of increasing concentrations of diSUMO2 or 4×SUMO2, respectively. Colour gradients for XRCC4 structures (PDB 1IK9) as indicated in (A). (**C**) Mutating XRCC4 regions implicated in SUMO2 binding in XRCC4’s head domain region abrogates 4×SUMO2 binding to XRCC4^1–164^ as assessed by biolayer interferometry (BLI). Vertical dashed lines separate association and dissociation phase. (**D**) mSUMO2 residues implicated in XRCC4^1–164^ (left) and PIAS2 (right) binding, as indicated by intensity losses of the ^1^H–^15^N HSQC spectra of mSUMO2 after addition of increasing concentrations of XRCC4^1–164^ or PIAS2 peptide (467-VDVIDLTIESS-477) (5). Colour gradients for mSUMO2 structures (PDB 2N1W) (76) as indicated in (A). (**E**) Photo-crosslinking of recombinant XRCC4^1–164^, incorporating *para*-benzoyl-phenylalanine (BpF) at R107, to 4×SUMO2. Crosslinked peptides in SUMO2-XRCC4^1–164^ bands highlighted by arrowhead on the left and identified by mass spectrometry (MS) are shown on the right. (**F**) As in (E) but for recombinant SUMO2 topologies incorporating BpF at R50, photo-crosslinked to recombinant XRCC4^1–164^. (**G**) Binding profiles of wildtype (WT) and mutant STMN1 (SIM43 residues mutated to alanines) to 4×SUMO2 by BLI. Vertical dashed lines separate association and dissociation phases.

Having narrowed down potential regions of SUMO binding to distinct and defined parts of XRCC4, we next performed NMR titrations of targeted XRCC4 truncations to map SUMO interactions at an increased—amino acid-level—resolution. To this end, we first established XRCC4^1–164^ as the largest head domain-containing XRCC4 construct amenable for NMR analysis (14). Two amino acid residue stretches showed marked intensity loss in the ^1^H–^15^N BEST-TROSY spectra, consistent with specific interaction between mSUMO2 and XRCC4 via discrete binding sites. Residues 101-LKDVSFRLGSF-111 displayed the strongest effects, followed by residues 56-ADDMA-60 (henceforth termed SIM101 and SIM56, respectively), with both regions forming coherent and spatially proximal surface sites on XRCC4’s head domain (Figure 5A; Supplementary Figure S2a,b), with a roughly estimated *K*_D_ in the high μM to low mM range. Equivalent experiments with diSUMO2 and 4×SUMO2 highlighted the same amino acid stretches (Figure 5B, Supplementary Figure S3a,b), further consolidating these regions as SUMO-binding surfaces on XRCC4. Additional affected residues likely reflect more extended contact regions due to the larger volumes occupied by the di-/4×SUMO2 topologies compared to mSUMO2. These findings confirmed our carbene footprinting results, highlighting peptide-level covalent fractional modification as a valuable technology for narrowing down interaction regions in an unbiased manner when little pre-existing information is available. In contrast to mSUMO2, addition of mSUMO1 resulted in fewer, different and substantially less pronounced changes in the XRCC4^1–164 1^H–^15^N BEST-TROSY spectra at the same equimolar titration ratios as those of mSUMO2. Most of the residues in the SIM56 and SIM101 regions were unaffected by the presence of mSUMO1, and only showed minor chemical shift perturbations rather than the pronounced intensity changes induced by mSUMO2 (Supplementary Fig S3c). Similarly, addition of XRCC4^1–164^ led to fewer, distinct and unsubstantial changes in the mSUMO1 ^1^H–^15^N BEST-TROSY spectra at the same equimolar titration ratios compared to mSUMO2 (Supplementary Figure S3d). Similar results were obtained for full-length XRCC4 (data not shown). The affected residues did not form discrete or coherent surface regions on XRCC4 (Supplementary Figure S3d). Together, these data demonstrated that binding of mSUMO1 to XRCC4 is substantially weaker than that of mSUMO2 and is non-specific in nature for XRCC4. In agreement with these findings, XRCC4 was preferentially co-precipitated by GST-mSUMO2 over GST-mSUMO1 in pulldown experiments (Supplementary Figure S3e). Collectively, these data demonstrate SUMO2 paralogue selectivity of XRCC4 for a specific site and on a protein-wide level.

The surface implicated in SUMO2 binding is negatively charged, with a positively charged patch on one side (Supplementary Figure S3f), differentiating this surface from that of established SIM classes (4,59). Consistent with our NMR analyses, individual or combined mutation of SIM56 and SIM101 abrogated binding of XRCC4^1–164^ to 4×SUMO2 (Figure 5C). In contrast to the pSIM mutants, mutation of SIM56 and SIM101 did not have marked destabilising effects on the overall structural integrity of the mutated proteins, as indicated by their proton NMR spectra (Supplementary Figure S4a) and aggregation onset temperature determination (Supplementary Figure S4b). Given their close spatial proximity, SIM56 and SIM101 likely synergise to form a split SIM that facilitates SUMO2 binding via a non-conventional paralogue-selective SUMO2-binding module on XRCC4’s head domain.

SUMO receptors can bind to different surfaces on SUMO1 and SUMO2 (5,59). To analyse how XRCC4-targeted SUMO2 surfaces correlate to the ones bound by other SUMO receptors, we compared the ^1^H–^15^N HSQC spectra of mSUMO2 in the absence and presence of increasing concentrations of XRCC4^1–164^. This revealed binding of XRCC4’s head to the β_2_/α_1_-groove on SUMO2, which is also targeted by other known SUMO receptors such as PIAS2 (5,6) (Figure 5D, Supplementary Figure S5a, b). Notably, several SUMO2 residues, affected by XRCC4 binding are not conserved in SUMO1, leading to alterations in charge (e.g. R36 in SUMO2 versus M40 in SUMO1) as well as in size (e.g. A23 in SUMO2 versus I27 in SUMO1; Supplementary Figure S5c), and resulting in charge distribution changes across the corresponding SUMO2/SUMO1 surfaces. For example, the relatively weak and evenly distributed positive charges on the XRCC4-bound SUMO2 surface (Supplementary Figure S5d) match the homogenously distributed negative charges on the reciprocal XRCC4 surface (Supplementary Figure S3f). By contrast, the equivalent SUMO1 surface possesses a strongly positively charged patch on one side (Supplementary Figure S5d), which could explain the unfavoured binding of SUMO1 to this XRCC4 region. Collectively, these analyses suggest that SUMO2 binding to XRCC4 is stabilised by ionic interactions that help achieve paralogue selectivity.

Given the unusual nature of the identified SUMO2:XRCC4 binding module, we performed photo-crosslinking experiments to further probe the binding surfaces. Recombinant XRCC4 incorporating the unnatural photo-crosslinkable amino acid *para*-benzoyl-phenylalanine (BpF) (16) at R107, proximal to SIM101, specifically crosslinked to the same β_2_/α_1_-groove of 4×SUMO2 after UV exposure (Figure 5E, Supplementary Figure S6a). Vice versa, various SUMO2 topologies integrating BpF at R50 (18), proximal to the identified XRCC4-binding region, also crosslinked specifically to SIM101 of XRCC4 (Figure 5F, Supplementary Figure S6b). Together, these findings consolidate the non-conventional nature of the identified SUMO-binding module on XRCC4’s head domain.

Finally, to test if this SUMO-binding module is conserved in other proteins, we screened the human proteome for motifs similar to SIM101 (K-[SDE]-[VLI]-[DES]-[FVLI]), identifying STMN1 as a potential candidate, which we had previously validated as a SUMO receptor in our screen (Figure 2B). Mutation of the XRCC4-like SIM101 residues (43-KDLSL-47, SIM43) to alanines markedly decreased binding to 4×SUMO2 (Figure 5G), confirming the conservation of XRCC4-like SIMs in other proteins. Moreover, amongst the >1000 proteins containing such sequences, known SUMO receptors were enriched, particularly when the XRCC4 SIM101-like residues were surrounded by acidic residues, suggesting the presence of supporting acidic residues for some of these SIMs, reminiscent of the acidic residues in SIM56 and their auxiliary role for XRCC4 SIM101.

### SUMO2 interaction of XRCC4’s head domain is incompatible with XLF binding

**Figure 6.**
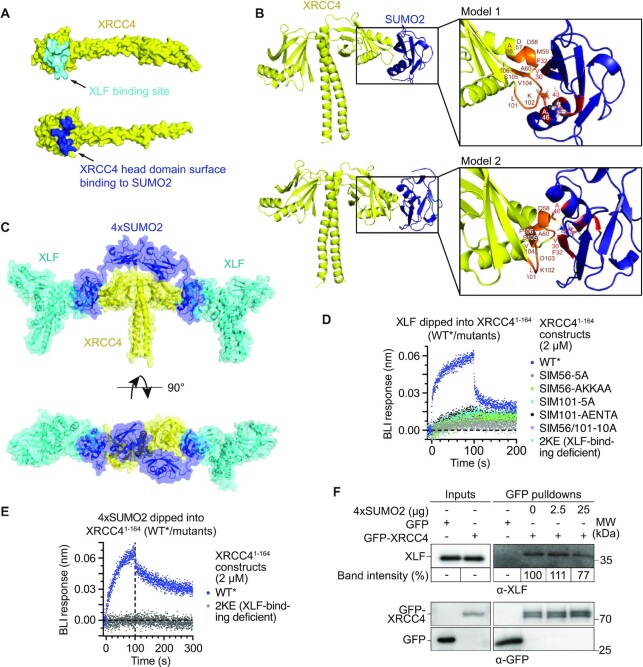
XRCC4 interaction with SUMO2 is incompatible with XLF binding. (**A**) XLF and SUMO2 binding to overlapping regions on XRCC4 (PDB 1FU1) (61). XLF-binding region (top) encompasses XRCC4 residues within 5 Å distance of L115 of XLF, based on XRCC4-XLF crystal structure (PDB 3RWR) (52). SUMO2-binding region entails XRCC4 residues 56–60 and 101–108. (**B**) HADDOCK models of 4×SUMO2 (PDB 2D07) (77) interaction with XRCC4 (PDB 1IK9) head domain. 56-ADDMA-60 and 101-LKDVSF-106 on XRCC4 and 30-VQFK-33, 40-LSKL-43 and A46 on SUMO2 highlighted in orange and red/pink, respectively, in models 1 and 2; polar interactions between D103 (XRCC4) and K42 (SUMO2) side chains in model 1, and D57 (XRCC4) and K42 (SUMO2) in model 2 highlighted with yellow dashed lines. (**C**) Crystal structure of XRCC4:XLF (PDB 3RWR) (52) overlaid with XRCC4:SUMO2 interaction model. (**D**) Biolayer interferometry (BLI) assays, illustrating abrogated XLF binding of XRCC4^1–164^ mutants. Vertical dashed line separates association and dissociation phases. (**E**) 4×SUMO2 binding of the 2KE XLF binding-deficient XRCC4^1–164^ mutant is abrogated in BLI assays. Vertical dashed line separates association and dissociation phases; BLI data for WT XRCC4^1–164^ are replicated from Figure 5C, forming part of the same experiment. (**F**) Precipitation of XLF by GFP-Trap pulldowns of GFP-XRCC4, ectopically expressed in HEK293T cells, from whole cell extracts after addition of increasing amounts of 4×SUMO2. Band intensities are averaged from two independent biological replicates and normalised to pulled-down XRCC4.

Because the SUMO2 interaction surface on the head domain of XRCC4 overlaps with XRCC4 binding to another NHEJ core factor, XLF (Figure 6A), we next investigated if SUMO2 and XLF binding were compatible. To help visualise the complex formation indicated by the NMR intensity losses, these losses were used to generate interaction restraints for molecular docking, using HADDOCK (15). The resulting structural models clustered into two orientations, both with several surface-exposed hydrophobic residues (L101, V104 and F106) of SIM101 located in the β6–β7 hairpin of XRCC4 stacked up with a hydrophobic patch centred on F32 on the reciprocal SUMO2 surface. The two orientations showed different stabilisation mediated by ionic interactions e.g. D103 (XRCC4) and K42 (SUMO2) in model 1, and D57 (XRCC4) with K42 (SUMO2) in model 2 (Figure 6B, Supplementary Figure S7a, Supplementary Table S4). Although not a truly rigid body docking, no substantive changes in the domains’ architectures resulted from the HADDOCK forcefield. The production of two orientations by the docking procedure may be a result of the limited number of restraints, or might indicate that both interactions take place in solution. The latter scenario is compatible with two SUMO2 moieties from the same polySUMO chain interacting with each head domain of the XRCC4 dimer, but in different orientations. We generated a structural model on this basis, and equilibrated it using molecular dynamics calculations via GROMACS (5 ns, 2 fs steps). Notably, no long-lived interactions were present in the simulations between the intervening SUMO2 moieties and XRCC4, consistent with the lack of NMR perturbations outside the major interaction surface formed by SIM56/SIM101. A 4×SUMO2 complex of this nature is consistent with the increased affinity of 4×SUMO2 for XRCC4, relative to mSUMO2. Both the mSUMO2 and 4×SUMO2 complexes are inconsistent with a tertiary complex including XLF (Figure 6C), which suggests competition between these proteins for XRCC4. Moreover, mutation of SIM56 and/or SIM101 abrogated XRCC4 binding to both SUMO2 (Figure 5C) and XLF (Figure 6D), similar to a known XLF binding-deficient mutant (53) (2KE: K65 and K99 mutated to glutamic acids; Figure 6D,E), without majorly affecting the overall structural integrity of the mutant, as indicated by its proton NMR spectrum (Supplementary Figure S7b) and aggregation onset temperature determination (Supplementary Figure S7c). In line with these findings, 4×SUMO2 showed a trend of competing with XRCC4-XLF interaction when added to whole cell extracts at 1 μM concentration (=25 μg) prior to GFP-XRCC4 pulldowns (Figure 6F). Altogether, these findings support a SUMO2-binding model that is incompatible with simultaneous binding of XLF to XRCC4.

### SUMO2 binding to XRCC4’s coiled-coil is incompatible with LIG4 interaction

**Figure 7.**
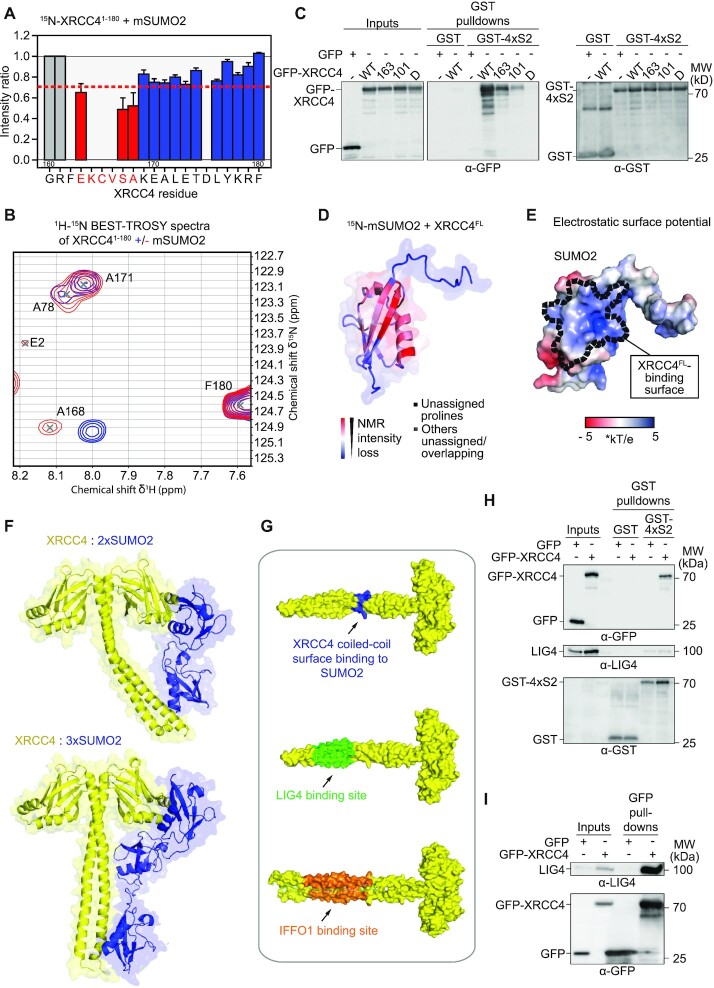
Non-conventional SUMO2-binding to XRCC4 coiled-coil overlaps with LIG4 and IFFO1 binding. (**A**) Intensity losses in the ^1^H–^15^N BEST-TROSY spectra of XRCC4^1–180^ after addition of increasing concentrations of SUMO2 monomers (mSUMO2). Error bars represent 1 standard deviation from the plotted value, as calculated from the noise levels in the TROSY spectra using the standard error propagation formula. Grey bars indicate residues with too low intensity for reliable measurement (arbitrarily set to 1); lack of bars represents unassigned residues. Red and blue bars indicate assigned residues in XRCC4^1–180^ and levels of intensity ratio loss of more or less than 30%, respectively. (**B**) ^1^H–^15^N BEST-TROSY spectra of XRCC4^1–180^ (red) showing key residues affected after addition of increasing concentrations of mSUMO2 (blue). (**C**) GST-4×SUMO2 pulldowns of GFP-XRCC4 WT, SIM101, SIM163 or double SIM101/SIM163 (D) mutants ectopically expressed in HEK293T XRCC4 KO cells. Pulldowns were repeated twice with similar results. S2: SUMO2. (**D**) Intensity losses in the ^1^H–^15^N HSQC spectra of mSUMO2 after addition of 0.33 molar equivalents of XRCC4^FL^. Colour gradient for mSUMO2 structure (PDB 2N1W) from red (most affected by binding) to blue (unaffected by binding). (**E**) Electrostatic surface potential of SUMO2 using APBS (78). Dashed line highlights key residues implicated in binding to XRCC4^FL^ according to (D). Electrostatic potential values are multiples of kT/e, kb: Boltzmann's constant, T: temperature (300 K); e: charge of an electron, conversion factor: 25.85 mV. (**F**) HADDOCK models of 2×SUMO2 (top) and 3×SUMO2 (bottom, based on PDB 2D07) interactions with XRCC4 (PDB 1IK9) head and coiled-coil domains. (**G**) Binding regions of SUMO2 (residues 163-EKCVSA-168; top), LIG4 (residues 173–19528; middle), and IFFO1 (residues 162–19630; bottom; PDB 6ABO30) on XRCC4 (PDB 1IK9). (**H**) GST pulldowns of GST and GST-4×SUMO2 with whole cell extracts obtained from HEK293T XRCC4 KO cells ectopically expressing GFP-XRCC4. (**I**) GFP-XRCC4 expressed in HEK293T cells co-precipitates a substantial fraction of total LIG4. Pulldowns were repeated twice with similar results. Inputs were 4% of the total.

In addition to SUMO2 binding to XRCC4’s head domain, our carbene footprinting and truncation studies pointed towards a SUMO2-binding region in the XRCC4 coiled-coil located in/around the 170–178 region. Having successfully assigned the majority of residues in XRCC4^1–164^, we extended our NMR analyses to XRCC4^1–180^. Taking the XRCC4^1–164^ assignment as a basis we were able to assign the majority of the additional 16 residues and detected intensity loss and/or perturbation shifts after addition of mSUMO2 for E163, S167 and A168 (Figure 7A, B), and possibly K164, C165 and V166, which could not be assigned, but were positioned in the centre of the affected residues (Figure 7A). Indeed, mutation of these residues (SIM163) to alanines reduced SUMO binding to XRCC4, albeit to a lesser extent than mutation of SIM101, with double mutation of SIM163 and SIM101 completely abrogating SUMO binding (Figure 7C), consistent with the carbene footprinting data. In contrast to SIM101 mutation, mutating SIM163 did not affect XLF binding, and LIG4 binding was unaffected in all SIM101/SIM163 single and double mutants (Supplementary Figure S7d). Interestingly, the interaction surface on SUMO2 did not markedly change based on intensity losses in the ^1^H–^15^N HSQC spectra of mSUMO2 after addition of full-length XRCC4 compared to XRCC4^1–164^ (compare Figure 7D and Supplementary Figure S7e to Figure 5D and Supplementary Figure S5a). We conclude that SIM56/SIM101 and SIM163 on the coiled-coil bind to comparable interaction surfaces on SUMO2 with similar surface charge profiles (compare Figure 7E to Supplementary Figure S5d). The presence of multiple SUMO-binding regions on XRCC4 opens the possibility for polySUMO2 chains not only crossing XRCC4’s head domain but spanning its head and coiled-coil domains, with a minimum of three individual SUMO moieties required for non-distortional binding (Figure 7F), providing an explanation for preferential binding of XRCC4 to longer SUMO topologies.

Strikingly, the affected coiled-coil region overlaps with XRCC4 binding to two other proteins important for NHEJ: LIG4 and IFFO1 (Figure 7G). Our model for SUMO-binding to XRCC4 SIM163 suggests that SUMO interaction in the coiled-coil region of XRCC4 is incompatible with XRCC4 interactions with LIG4 and IFFO1. To test this hypothesis, we performed GST–4×SUMO2 pulldown assays, demonstrating that XRCC4, but not LIG4, could be co-precipitated from whole cell extracts (Figure 7H), while LIG4 was successfully co-precipitated from whole cell extracts in GFP-XRCC4 pulldowns (Figure 7I). Collectively, these data indicate that LIG4 and polySUMO2 binding to XRCC4 are incompatible and similar principles likely apply to IFFO1 binding. This does not necessarily mean that 4×SUMO2 prevents XRCC4 binding to LIG4 or IFFO1. Instead, or in addition, 4×SUMO2 binding to XRCC4 may affect functions of XRCC4 that are not related to LIG4-/IFFO1–XRCC4 complexes, with a fraction of XRCC4 having been shown to exist in cells without for example being bound to LIG4 (10).

### XRCC4 SIM101 plays a role in non-homologous end-joining

**Figure 8.**
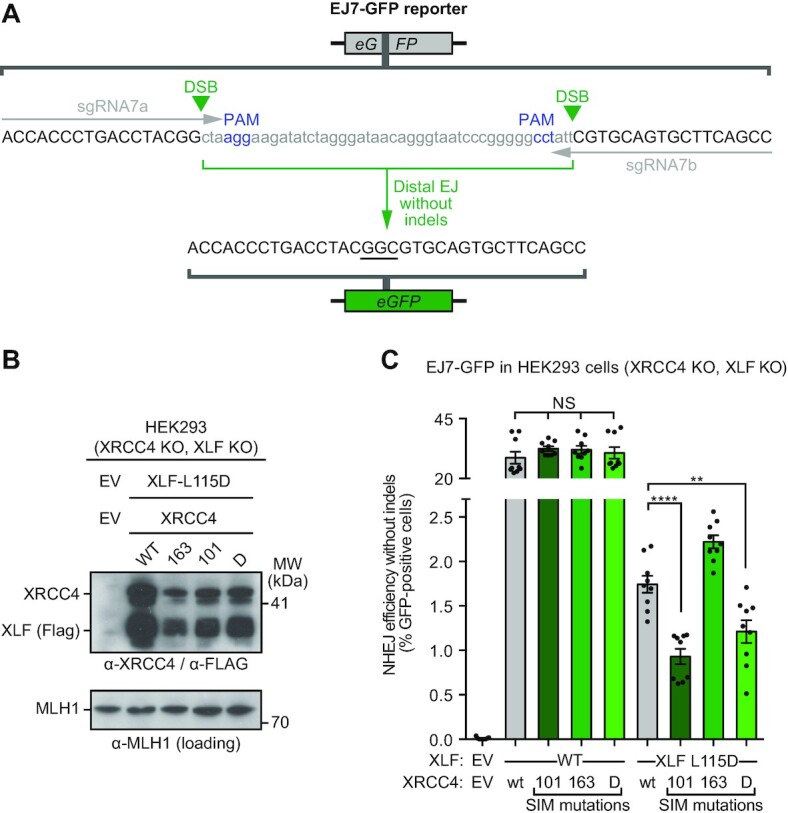
XRCC4 SIM101 promotes non-homologous end-joining (NHEJ). (**A**) Schematic of EJ7-GFP reporter assay (66). (**B**) Immunblots showing complementation levels of XLF and XRCC4 proteins (wildtype (WT), SIM101-5A single, SIM163-5A single, and SIM101/163-10A double (D) mutants) in HEK293 XLF/XRCC4 double KO cells, stably integrating EJ7-GFP. (**C**) XRCC4’s SIM101 but not SIM163 is important for DNA double-strand break (DSB) repair by NHEJ, specifically distal end-joining (EJ) of broken DNA ends without insertions/deletions (indels). EV: empty vector; 101: SIM101-5A mutant; 163: SIM163-5A mutant; D: SIM101/163-10A double mutant. *P* values are based on Mann–Whitney analyses (NS: not significant, *P*>0.05; **: *P*=0.0056; ****: *P*<0.0001).

To assess if the SUMO:XRCC4 interaction plays a role in NHEJ, we used a recently established GFP reporter platform (EJ7-GFP) that measures the efficiency of XRCC4-dependent distal end-joining of broken DSB ends without indels (Figure 8A) (66). To do so, we initially measured NHEJ efficiencies in HEK293 XRCC4 KO cells stably integrating the EJ7-GFP reporter. We complemented the cells with XRCC4—WT, SIM101 or SIM163, singly or doubly mutated. All XRCC4 SIM mutants fully complemented the NHEJ efficiencies to WT levels, indicating potential redundancies of the assessed SIMs (data not shown). Given that the SIM101 region also mediates binding to XLF, an NHEJ core factor known for its redundancy in NHEJ, we wanted to test if any of the XRCC4 SIMs were redundant with certain aspects of XLF function. To assess this, we generated a HEK293 XRCC4/XLF double KO cell line, stably integrating the EJ7-GFP reporter system (Supplementary Figure S7f). While the immunoblot signals from each of the mutants were slightly reduced compared to XRCC4 WT, each of the mutants was detected at similar levels (Figure 8B). By contrast, the mutants were distinct in their abilities to promote NHEJ. Complementation with WT XLF and XRCC4—WT, SIM101 or SIM163, singly or doubly mutated—reconstituted NHEJ efficiencies to similar levels (Figure 8C), confirming our initial observations. By contrast, complementation with XRCC4—SIM101 singly or SIM101/SIM163 doubly (D) mutated—together with XLF L115D, a well-established XLF mutant unable to bind XRCC4, and diminished in its XRCC4/LIG4-stimulating capacity (53,67), was significantly less efficient (∼2-fold) in reconstituting NHEJ efficiency (Figure 8C). Interestingly, XRCC4, singly mutated for SIM163, did not show any reductions in NHEJ efficiency in these settings (Figure 8C). Collectively, these findings indicate that SIM101, but not SIM163, is important for promoting NHEJ in circumstances when certain aspects of XLF are compromised.

## DISCUSSION

SUMOylations affect thousands of proteins regulating a gamut of cellular processes, but only tens of SUMO receptors decoding these SUMOylations have been validated. Using human proteome microarrays with fluorescently labelled SUMO topologies, we uncover >200 new binary polySUMO2/3 receptor candidates and validate a substantial fraction of them, markedly extending our known human SUMO receptor pool. Given the involvement of the identified receptors in diverse cellular pathways, these results serve as a platform for breaking new ground in SUMO biology. Indeed, numerous further opportunities now await exploration to uncover mechanisms underlying established as well as under-studied areas of SUMO biology, including epigenetics, pre-mRNA splicing, transcriptional regulation, DNA repair, cytoskeleton organisation and protein synthesis in health and disease.

Our screening pipeline is widely applicable to ubiquitin and other UBLs. By demonstrating its utility for paralogue- and topology-selective receptor discovery for polySUMO2, we provide a paradigm for narrowing down ubiquitin/UBL-binding regions for proteins lacking conventional binding modules, using a recently developed structural mass spectrometry technique—carbene footprinting—that can be applied independently of protein size, quantity of starting material, and interaction affinity, factors commonly limiting high-resolution analytical methodologies (12,13,65).

By combining carbene footprinting and mutational and structural analyses with genetic code expansion, we characterise XRCC4 as the first core NHEJ factor containing non-conventional SUMO-binding regions, revealing two distinct SUMO-binding modules along its sequence. NHEJ has long remained under-studied for its regulation by SUMOylation compared to other DSB and DNA repair pathways (9). Only recently—during the course of our work—has another study explored XRCC4 as a SUMO receptor focussing on pSIM33 and its neighbouring W43 as the relevant SUMO-binding region. The authors propose that disrupted SUMO binding contributes to the pathogenesis of the XRCC4 W43R patient mutation (35). However, W43 and L36 along with several other residues make up the hydrophobic core of XRCC4’s head domain, consistent with extensive hydrogen exchange protection in this area (14). As such, these residues are surface-inaccessible (14) and critical for XRCC4’s structural integrity and stability, consistent with the markedly decreased aggregation onset temperature we observed for the pSIM33 mutant (Supplementary Figure S1e). Indeed, XRCC4 protein levels are substantially reduced in W43R patient cells (68), making this scenario and the direct relevance of this region for SUMO binding highly unlikely.

Intriguingly, the XRCC4 SUMO-binding module we identified on its head domain features paralogue selectivity for SUMO2/3 over SUMO1, and as a whole, XRCC4 preferentially bound to polySUMO2 chains over shorter topologies. While SUMO binding relies on a hydrophobic patch in line with conventional SIMs, a positive charge at the core of the SUMO-binding module, K102, represents an unprecedented characteristic for this binding. Given the importance of acidic residues for mediating interactions with SUMO1 (5), this positive charge along with additional characteristics likely contributes to the paralogue selectivity we observed.

Despite the surprising features of XRCC4’s SUMO-binding surface, the reciprocal region on SUMO2 was similar to the one targeted by other SUMO receptors, albeit with different nuances in the precise residue involvement. These data highlight the versatility of SUMO to utilise the same region for interactions with a wide range of distinct SUMO-binding modules.

Notably, XRCC4-like SIM features are enriched in known SUMO receptors and can be detected in a large number of proteins overall. Indeed, we showed XRCC4 SIM101-like SUMO binding in another SUMO receptor identified in our screen, raising the possibility that this type of binding module contributes to SUMO decoding across wide and diverse areas in cell biology. Together with validating a receptor lacking both conventional and XRCC4-like SIMs—TCEAL6—our results suggest an unanticipated spectral plasticity of SUMO-binding modules that has remained undiscovered, and for which our screen of binary SUMO receptors now provides a rich resource.

Mechanistically, the two SUMO-binding regions on XRCC4 overlap or involve common features with other XRCC4-interaction regions important for NHEJ. The use of common interaction sites has emerged as an intriguing concept to make efficient use of a limited number of binding sites available on NHEJ core factors to provide functional redundancy. In line with this notion, we find that XRCC4’s SIM101 becomes important for promoting NHEJ when certain features of XLF are impaired. How exactly SIM101 contributes to NHEJ under these circumstances, perhaps by stabilising certain NHEJ complexes, their conformations and/or their activity, will be interesting topics to address in future investigations. In this regard, it is noteworthy that only one of the SUMO-binding modules, SIM101, but not SIM163, participated in this function, raising the possibility that different XRCC4 SIMs and/or their combinations might be implicated in distinct aspects of NHEJ regulation and/or associated pathways, perhaps by recognising different SUMOylated substrates. In such circumstances, the identified interaction sites could lead to diversity that can help cells deal with different types of DNA damage arising in distinct chromatin contexts and with varying sets and/or levels of functional repair factors. Such scenarios could apply to, and be relevant for, different tissues and developmental stages, as well as in different cancer settings due to differential regulation of NHEJ factors, their downregulation and/or their dysfunctioning.

Linking common binding sites to recognition of SUMOylations occurring in a spatiotemporally regulated manner such as in response to DNA damage (9), could help cells coordinate the use of common binding regions in an optimal manner, enabling them to target distinct repair complexes to the most appropriate types of DSBs in varying chromatin environments and at different repair stages, while preventing harmful competition between them. Our findings provide possible future avenues for exploring the mechanistic basis of these processes, which remain a puzzling phenomenon in NHEJ. Another possibility is that XRCC4 interactions with SUMO negatively regulate NHEJ by disrupting or preventing interactions with XLF, LIG4 and IFFO1. Such a mechanism could complement the recently demonstrated NHEJ barrier mediated by RIG1, which blocks XRCC4 interactions with XLF and LIG4 to counteract viral integration (69). Given the known involvement of SUMO in viral defence mechanisms, our work provides an attractive lead to explore such regulatory layers in future investigations. Alternatively, disruption of XRCC4 complexes after completion of repair could be important for finalizing NHEJ, in analogy to the release of Ku after repair has taken place (70,71,72), and in that way contribute to other mechanisms negatively regulating XRCC4 interactions (50,73).

Finally, we note that our work may have medical applications because targeting DDR and ubiquitin/UBL system components can be exploited to treat cancer. Indeed, targeting NHEJ at the level of XRCC4 interactions represents an attractive approach to sensitise cancer cells, commonly displaying cryptic DNA repair pathway defects including NHEJ, via synthetic lethality and/or other mechanisms (74). Similarly, given the importance of DSB repair pathway choice for determining CRISPR-Cas9 genome editing outcomes, targeting specific XRCC4 interactions important for NHEJ may also be relevant for increasing the efficiency of precise gene editing processes relying on homology-dependent repair.

## DATA AVAILABILITY

Assignments of XRCC4^1-180^ residues 167–180 are deposited with BMRB code 50742. PDB files for the different XRCC4:SUMO models are available as supplementary files. Other original data are available upon request.

## Supplementary Material

gkac237_Supplemental_Files
