# Supplementary material for: Microarray screening reveals two non-conventional SUMO-binding modules linked to DNA repair by non-homologous end-joining
Source: Nucleic Acids Res. 2022 Apr 14;50(8):4732–54. doi: 10.1093/nar/gkac237 (PMC9071424; doi:10.1093/nar/gkac237)
Supplement: gkac237_Supplemental_Files [file gkac237_supplemental_files.zip › Supplementary Figures plus captions.pdf]

Figure S1

a

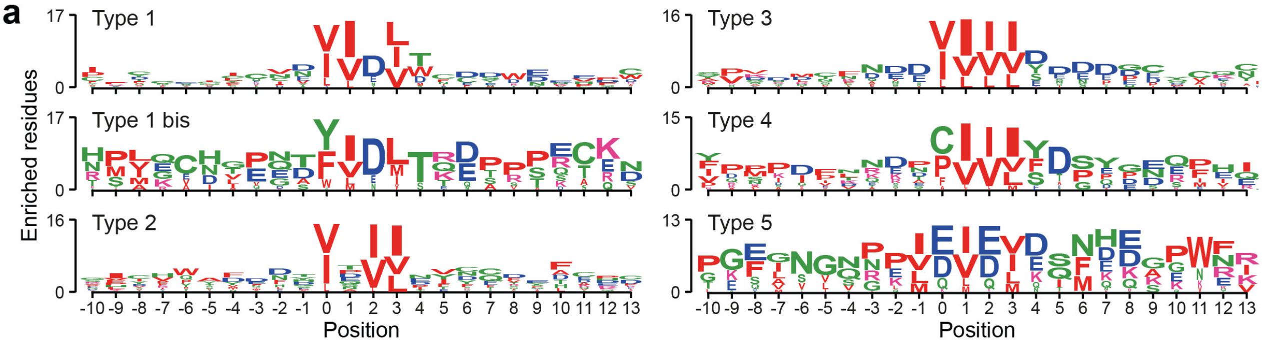

b

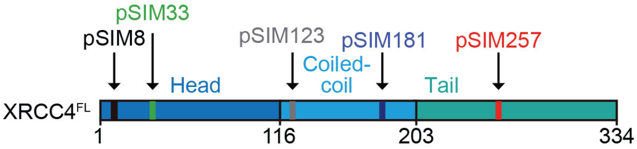

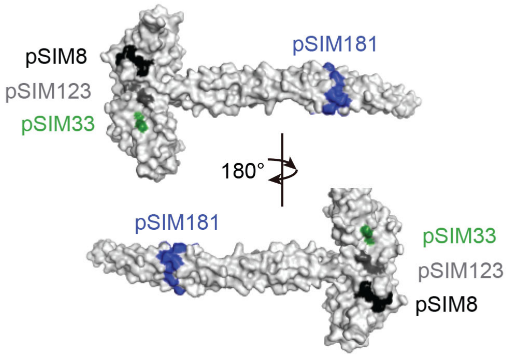

Putative SIM (GPS-SUMO)	Position	Sequence		
pSIM8	8-12	MERKISR	IHLVS	EPSITHF
pSIM33	33-37	KTLESGF	VITLT	DGHSAWT
pSIM123	123-127	VENPAEV	IRELI	CYCLDTI
pSIM181	181-185	TDLYKRF	ILVLN	EKKTKIR
pSIM257	257-261	AVSKDDS	IISSL	DVTDIAP

c

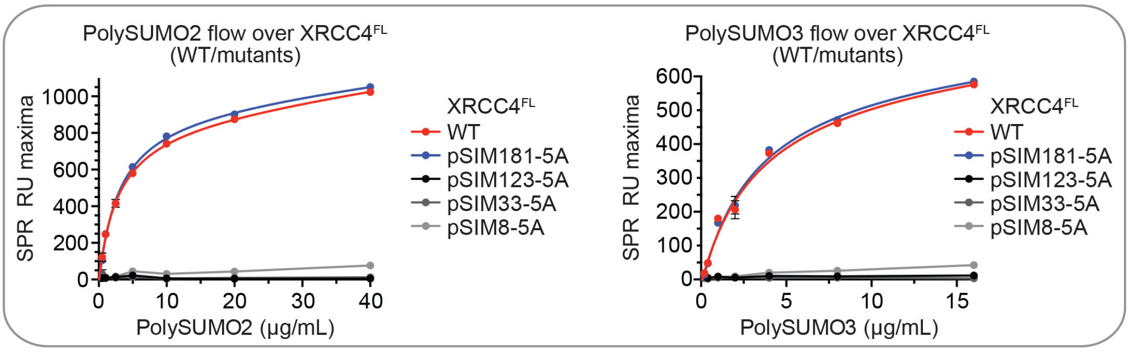

d

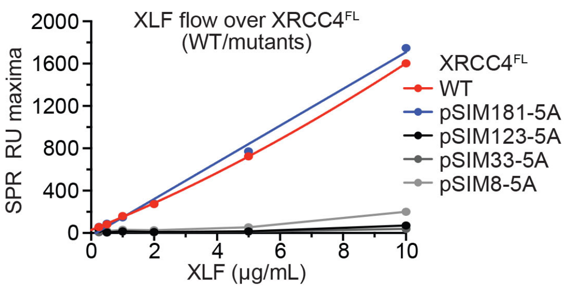

e

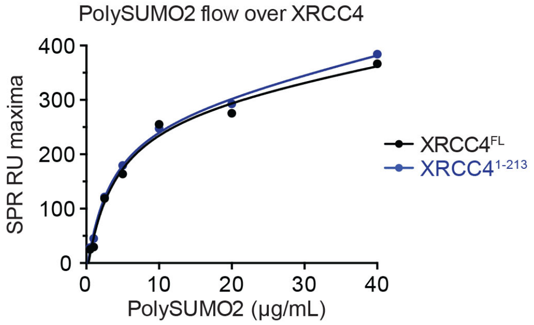

f

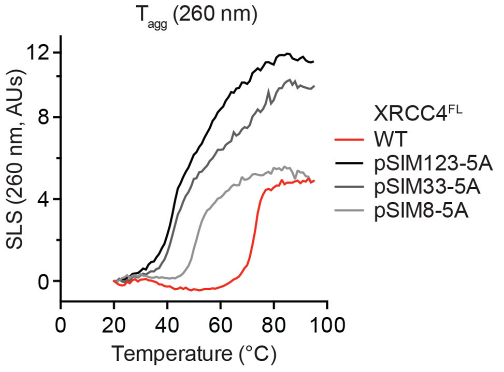

$XRCC4^{FL}$	T_{agg} (260 nm)
WT	67.50 °C
pSIM8	45.67 °C
pSIM33	36.84 °C
pSIM123	37.05 °C

Supplementary Figure S1. XRCC4 binding to SUMO2 is independent of conventional SUMO interacting motifs (SIMs). (**a**) Consensus motifs for conventional SUMO-interacting motifs (SIMs) according to JASSA, created using PSSMSearch (78). (**b**) Schematic of full-length XRCC4 ($XRCC4^{FL}$) on the top, highlighting four of five putative SIMs (pSIMs) predicted in silico using JASSA and GPS-SUMO. Underscores in the pSIM sequences indicate residues forming extensive interactions with nearby XRCC4 residues, or residues buried deep inside the head domain, as shown in the $XRCC4^{1-164}$ structure at the bottom (PDB 1IK9; colour code according to pSIMs). (**c**) Equilibrium analysis of SPR response unit maxima of polySUMO2 (left) and polySUMO3 (right) binding to immobilised $XRCC4^{FL}$ wildtype (WT) and mutant versions, with their pSIM residues mutated to alanines (pSIM-5A mutants). (**d**) Equilibrium analysis of SPR response unit maxima of XLF binding to immobilised $XRCC4^{FL}$, WT and pSIM-5A mutants. (**e**) Equilibrium analysis of SPR response unit maxima of polySUMO2 binding to immobilised $XRCC4^{FL}$ and $XRCC4^{1-213}$. (**f**) Protein stability determination based on aggregation temperatures (T_{agg}'s) calculated from static light scattering (SLS) profiles measured over a defined temperature ramp as indicated. Note that SLS profiles after the start of aggregation can vary due to possible precipitation of the protein. RU: response unit. SPR: surface plasmon resonance.

Figure S2

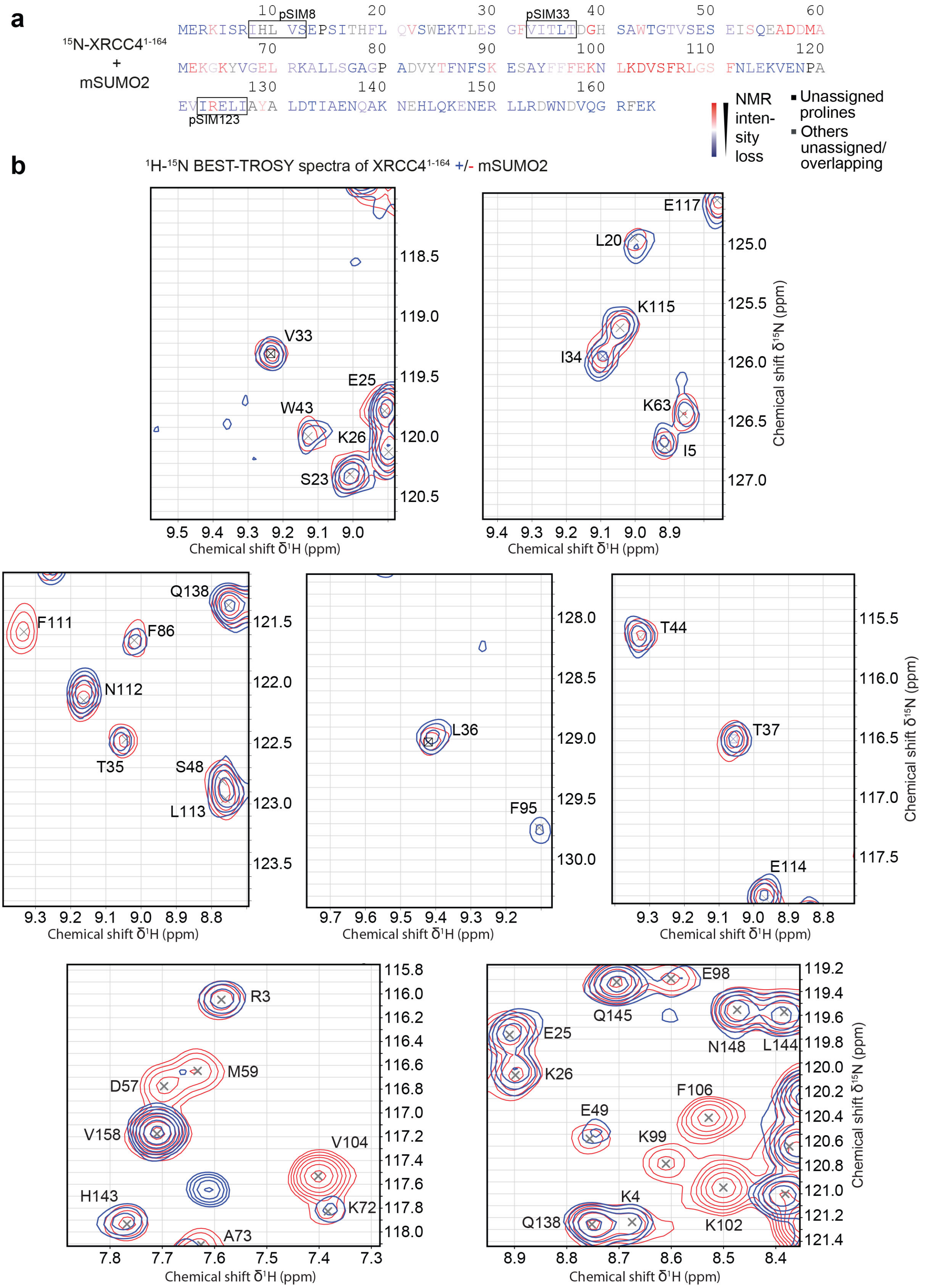

a
15N-XRCC41-164 + mSUMO2
MERKISRIHL VSEPSITHFL QVSWEKTLES GFVITLTDGH SAWTGTVSES EISQEADDMA
MEKGKYVGEL RKALLSGAGP ADVYTFNFSK ESAYFFFEKN LKDVSFRLGS FNLEKVENPA
EVIRELIAYA LDTIAENQAK NEHLQKENER LLRDWNDVQG RFEK
pSIM8
pSIM33
pSIM123
NMR intensity loss
Unassigned prolines
Others unassigned/ overlapping
b
1H-15N BEST-TROSY spectra of XRCC41-164 +/- mSUMO2
Chemical shift δ1H (ppm)
Chemical shift δ15N (ppm)
V33
E25
W43
K26
S23
E117
L20
K115
I34
K63
I5
Q138
F111
F86
N112
T35
S48
L113
L36
F95
T44
T37
E114
R3
M59
D57
V158
V104
H143
K72
A73
E98
Q145
N148
L144
F106
E49
K99
K4
K102

Supplementary Figure S2. XRCC4$^{1-164}$ residues implicated in binding to SUMO2 monomers. (**a**) XRCC4$^{1-164}$ residues implicated in SUMO2 monomer (mSUMO2) binding, as indicated by intensity losses in the ^{1}H-^{15}N BEST-TROSY spectra of XRCC4$^{1-164}$ after addition of increasing concentrations of mSUMO2. Colour gradient for XRCC4 residues ranges from red (most affected by binding) to blue (unaffected by binding). Unassigned prolines shown in black, other unassigned/overlapping residues in grey. Note the lack of or minimal responses present in the conventional putative SIMs (pSIMs) highlighted by boxes. (**b**) Representative panels of key interacting residues compared to pSIM residues of the XRCC4$^{1-164}$ ^{1}H-^{15}N HSQC spectra (red) overlayed with the XRCC4$^{1-164}$ spectra after the addition of 0.25 equivalents of SUMO2 monomer (mSUMO2) (blue).

Figure S3

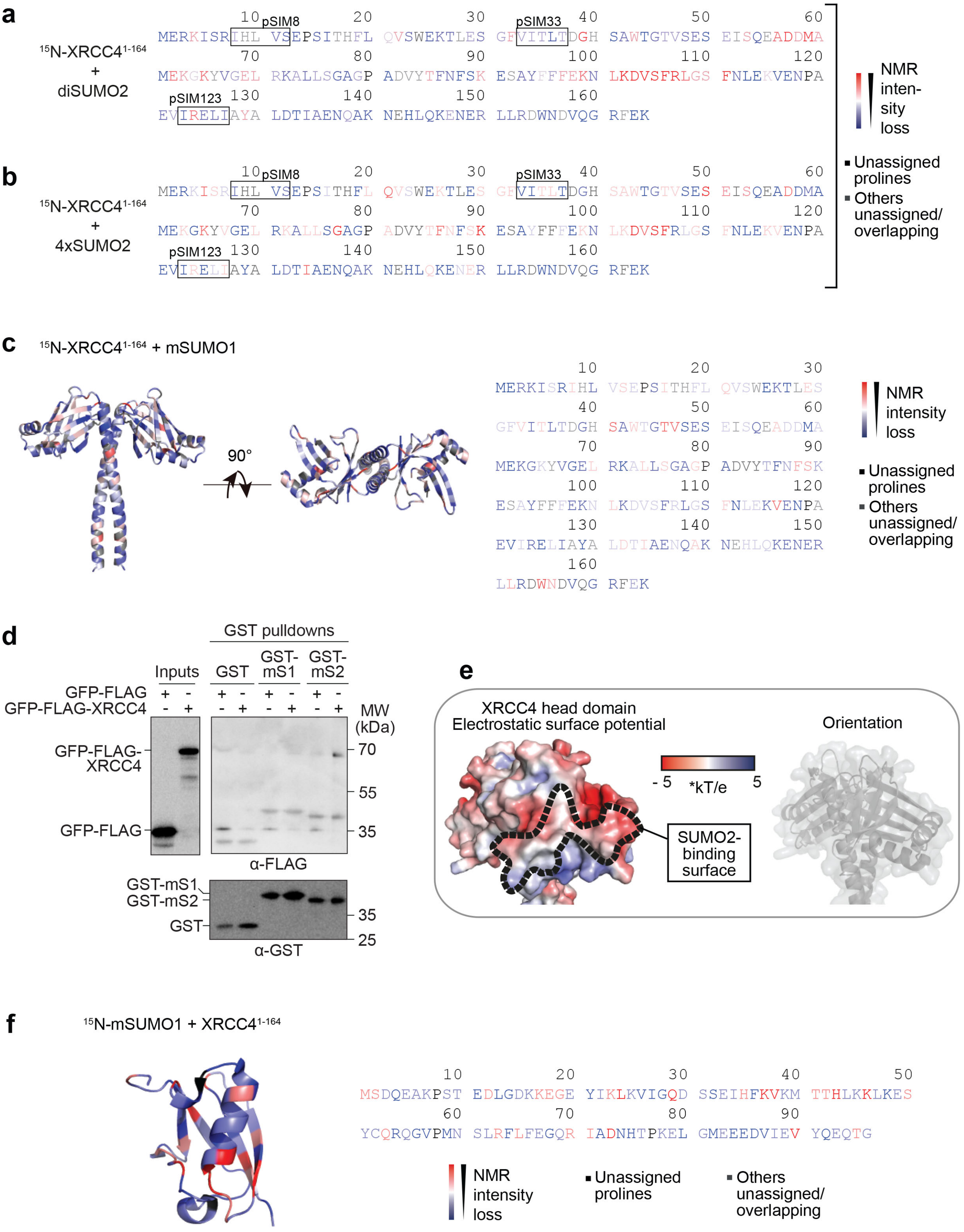
a
15N-XRCC4 1-164 + diSUMO2
pSIM8
pSIM33
pSIM123
MERKISRIHL VSEPSITHFL QVSWEKTLES GFVITLTDGH SAWTGTVSES EISQEADDMA
MEKGKYVGEL RKALLSGAGP ADVYTFNFSK ESAYFFFEKN LKDVSFRLGS FNLEKVENPA
EVIRELIAYA LDTIAENQAK NEHLQKENER LLRDWNDVQG RFEK
b
15N-XRCC4 1-164 + 4xSUMO2
NMR intensity loss
Unassigned prolines
Others unassigned/ overlapping
c
15N-XRCC4 1-164 + mSUMO1
90°
d
GST pulldowns
Inputs
GST
GST-mS1
GST-mS2
GFP-FLAG
GFP-FLAG-XRCC4
MW (kDa)
70
55
35
α-FLAG
GST-mS1
GST-mS2
GST
25
α-GST
e
XRCC4 head domain
Electrostatic surface potential
-5
*kT/e
5
SUMO2-binding surface
Orientation
f
15N-mSUMO1 + XRCC4 1-164
MSDQEAKPST EDLGDKKEGE YIKLKVIGQD SSEIHFKVKM TTHLKKLKES
YCQRQGVPMN SLRFLFEGQR IADNHTPKEL GMEEEDVIEV YQEQTG

Supplementary Figure S3. XRCC4$^{1\text{-}164}$ residues implicated in binding to different SUMO topologies. XRCC4$^{1\text{-}164}$ residues implicated in SUMO2 binding, as indicated by intensity losses in the ^{1}H-^{15}N BEST-TROSY spectra of XRCC4$^{1\text{-}164}$ after addition of increasing concentrations of SUMO2 dimers (diSUMO2) (**a**), SUMO2 tetramers (4xSUMO2) (**b**), or SUMO1 monomers (mSUMO1). (**c**) Colour gradient for XRCC4 residues ranges from red (most affected by binding) to blue (unaffected by binding). Unassigned prolines shown in black, other unassigned/overlapping residues in grey. Boxes highlight putative conventional SIMs. (**d**) GST-pulldowns of SUMO1 (mS1) and SUMO2 (mS2) monomers with whole cell extracts of HEK293T cells, ectopically expressing GFP-FLAG or GFP-FLAG-XRCC4. Note that the anti-FLAG antibody led to some unspecific detection of GST, GST-mS1 and GST-mS2 in the pulldowns. (**e**) Electrostatic surface potential of XRCC4 head domain (PDB 1IK9), with key residues involved in SUMO2 binding (56-ADDMA-60, 101-LKDVS-105) highlighted inside dashed line; created using APBS electrostatics plugin for Pymol (77). Positively charged surfaces are shown in blue, neutral ones in white, negatively charged in red, with values displayed as multiples of kT/e: kb: Boltzmann's constant, T: temperature n (300 K); e: charge of an electron; conversion factor: 25.85 mV. (**f**) mSUMO1 residues implicated in XRCC4$^{1\text{-}164}$ binding, as indicated by chemical shift perturbations in the ^{1}H-^{15}N HSQC spectra of 1 mSUMO1 equivalent of XRCC4$^{1\text{-}164}$. Colour gradient: red (residues most affected by binding) to blue (residues unaffected by binding). Unassigned prolines displayed in black, other unassigned/overlapping residues in grey. Detailed view of key mSUMO1 residues affected is shown on the right.

Figure S4

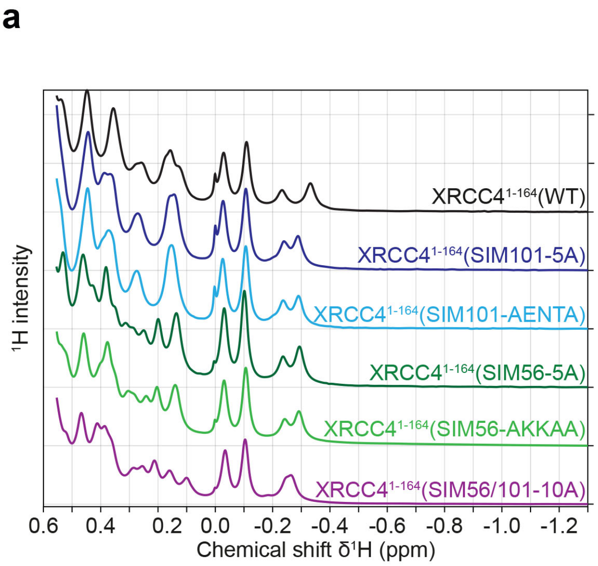

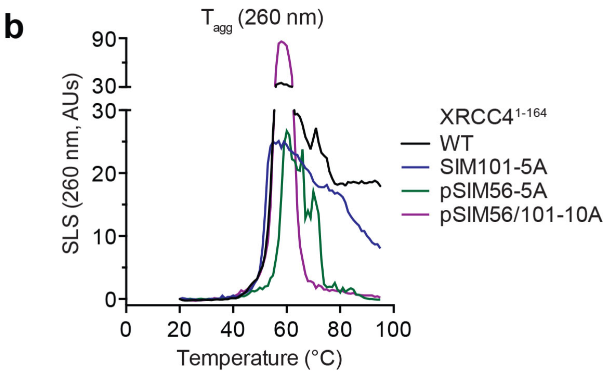

**XRCC4$^{1-164}$**	**T_{agg} (260 nm)**
WT	49.31 °C
SIM101-5A	48.55 °C
SIM56-5A	53.08 °C
SIM56/101-10A	52.76 °C

Supplementary Figure S4. No marked destabilising effects of SIM56 and SIM101 mutations on XRCC4 fold. (**a**) 1D ^{1}H NMR spectra of wildtype (WT) and the indicated mutant XRCC4$^{1-164}$ constructs, showing the methyl regions of the spectra, which are extremely sensitive to structural perturbations, as the chemical shifts are defined by the angular and distance relationships with aromatic rings, which are fixed in the folded regions of proteins. Note that some resonances changed because methyl-containing residues have been substituted. (**b**) Protein stability determination based on aggregation temperatures (T_{agg}'s) calculated from static light scattering (SLS) profiles measured over a defined temperature ramp as indicated. Note that SLS profiles after the start of aggregation can vary due to possible precipitation of the protein.

Figure S5

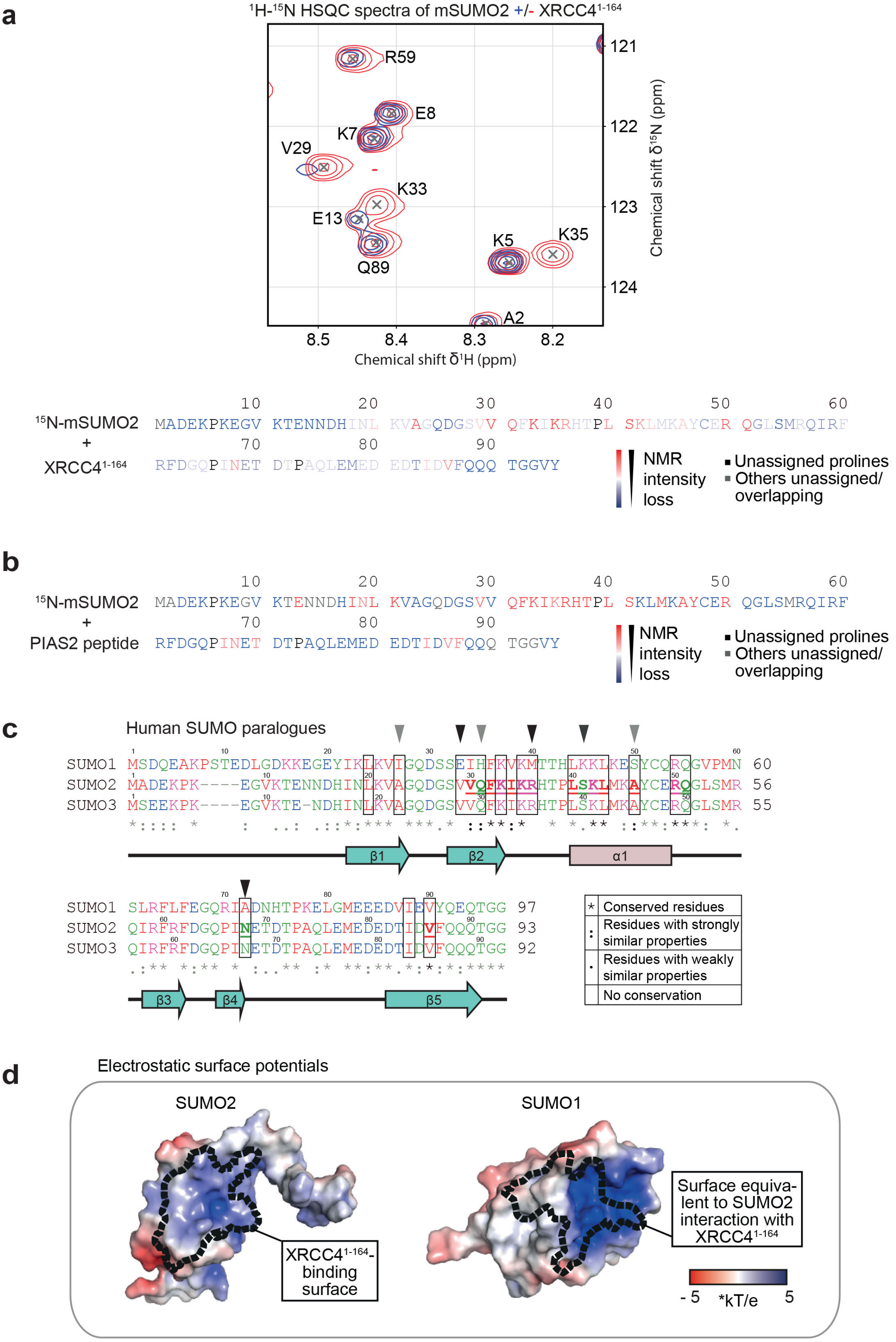

a
1H-15N HSQC spectra of mSUMO2 +/- XRCC4 1-164
R59
E8
K7
V29
K33
E13
Q89
K5
K35
A2
121
122
123
124
8.5
8.4
8.3
8.2
Chemical shift δ1H (ppm)
Chemical shift δ15N (ppm)
15N-mSUMO2
+
XRCC4 1-164
10 20 30 40 50 60
MADEKPKEGV KTENNDHINL KVAGQDGSVV QFKIKRHTPL SKLMKAYCER QGLSMRQIRF
70 80 90
RFDGQPINET DTPAQLEMED EDTIDVFQQQ TGGVY
NMR intensity loss
Unassigned prolines
Others unassigned/ overlapping
b
15N-mSUMO2
+
PIAS2 peptide
10 20 30 40 50 60
MADEKPKEGV KTENNDHINL KVAGQDGSVV QFKIKRHTPL SKLMKAYCER QGLSMRQIRF
70 80 90
RFDGQPINET DTPAQLEMED EDTIDVFQQQ TGGVY
NMR intensity loss
Unassigned prolines
Others unassigned/ overlapping
c
Human SUMO paralogues
SUMO1 MSDQEAKPSTEDLGDKKEGEYIKLKVIGQDSSEIHFKVKMTTHLKKLKESYCQRQGVPMN 60
SUMO2 MADEKPK----EGVKTENNDHINLKVAGQDGSVVQFKIKRHTPLSKLMKAYCERQGLSMR 56
SUMO3 MSEEKPK----EGVKTE-NDHINLKVAGQDGSVVQFKIKRHTPLSKLMKAYCERQGLSMR 55
β1
β2
α1
SUMO1 SLRFLFEGQRIADNHTPKELGMEEEDVIEVYQEQTGG 97
SUMO2 QIRFRFDGQPINETDTPAQLEMEDEDTIDVFQQQTGG 93
SUMO3 QIRFRFDGQPINETDTPAQLEMEDEDTIDVFQQQTGG 92
β3
β4
β5
* Conserved residues
: Residues with strongly similar properties
. Residues with weakly similar properties
No conservation
d
Electrostatic surface potentials
SUMO2
SUMO1
XRCC4 1-164-binding surface
Surface equivalent to SUMO2 interaction with XRCC4 1-164
- 5
*kT/e
5

Supplementary Figure S5. XRCC4-binding region on SUMO2 and comparative SUMO paralogue features. (**a**) Top: representative panel of mSUMO2 ^{1}H-^{15}N HSQC spectra displaying key residues affected without (red) and with (blue) 0.25 equivalents of XRCC4$^{1\text{-}164}$ added. Bottom: SUMO2 residues implicated in XRCC4$^{1\text{-}164}$ as indicated by intensity losses in the ^{1}H-^{15}N BEST-TROSY spectra of monomeric SUMO2 (mSUMO2) after addition of increasing concentrations of XRCC4$^{1\text{-}164}$. Colour gradient for SUMO2 residues ranges from red (most affected by binding) to blue (unaffected by binding). Unassigned prolines shown in black, other unassigned/overlapping residues in grey. (**b**) ^{1}H-^{15}N HSQC spectra like described in (a) but for PIAS2 peptide (467-VDVIDLTIESS-478). (**c**) Sequence alignment of mature forms of human SUMO1, SUMO2 and SUMO3 paralogues using Clustal Omega (79). Secondary structure elements are depicted below the sequence alignment. (**d**) Electrostatic surface potentials of SUMO2 and SUMO1. Left: dashed lines highlight key residues of SUMO2 implicated in binding to XRCC4$^{1\text{-}164}$ according to residues boxed in SUMO2 in (c). Right: equivalent SUMO1 residues boxed in (c). Electrostatic potential generated using APBS electrostatics plugin for Pymol (77), with negatively and positively charged values ranging from red to blue, respectively, and displayed as multiples of kT/e, kb: Boltzmann's constant, T: temperature (300 K); e: charge of an electron; conversion factor: 25.85 mV.

Figure S6

a

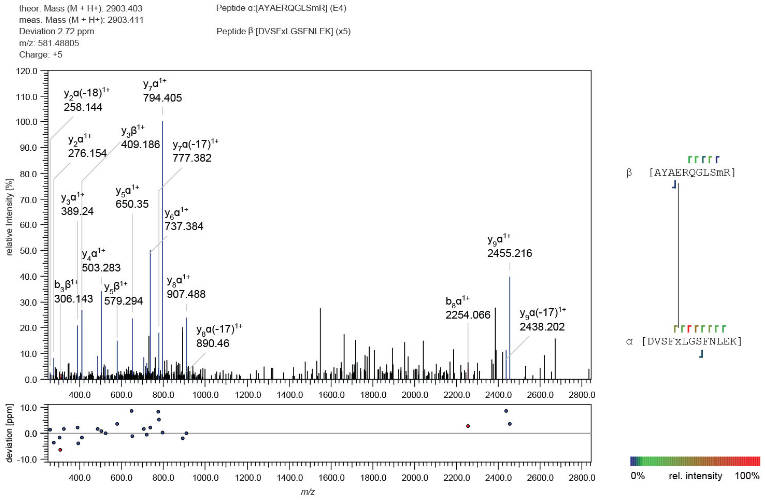

XRCC4$^{1-164}$ R107BpF + 4xSUMO2 (upper band)

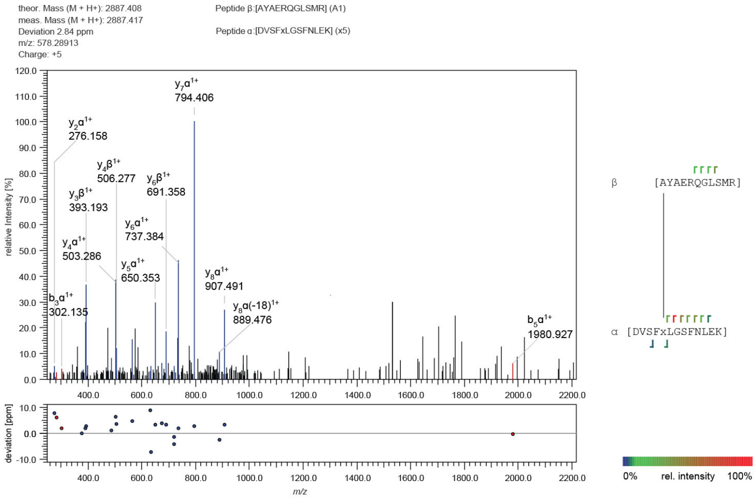

XRCC4$^{1-164}$ R107BpF + 4xSUMO2 (lower band)

b

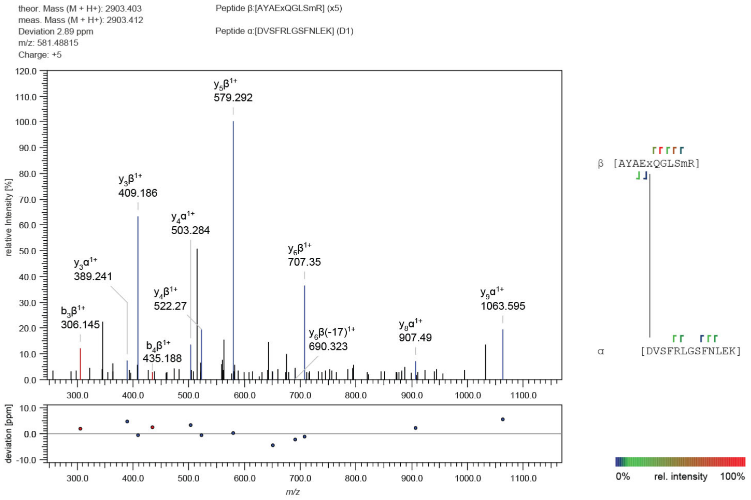

4xSUMO2 R50BpF+ XRCC4$^{1-164}$

Supplementary Figure S6. SUMO:XRCC4$^{1\text{-}164}$ photo-crosslinking maps interaction region to XRCC4's SIM101 and SUMO2's β2/α1-groove. (**a**) Mass spectra of cross-linked peptides derived from upper (top) and lower (bottom) gel bands of photocrosslinked products between recombinant XRCC4$^{1\text{-}164}$ incorporating para-benzoyl-phenylalanine (BpF) at R107 and recombinant 4xSUMO2. Crosslinking patterns for the upper and lower crosslinked gel bands are the same, with examples for crosslink positions shown on the right. (**b**) Mass spectrum of cross-linked peptides derived from gel band of photocrosslinked products between recombinant 4xSUMO2 incorporating BpF at R50 and recombinant XRCC4$^{1\text{-}164}$. Similar crosslinking patterns were retrieved with di- or monoSUMO2 incorporating BpF at R50. Examples for crosslink positions are shown on the right. Small 'm' symbolises oxidised methionine.

Figure S7

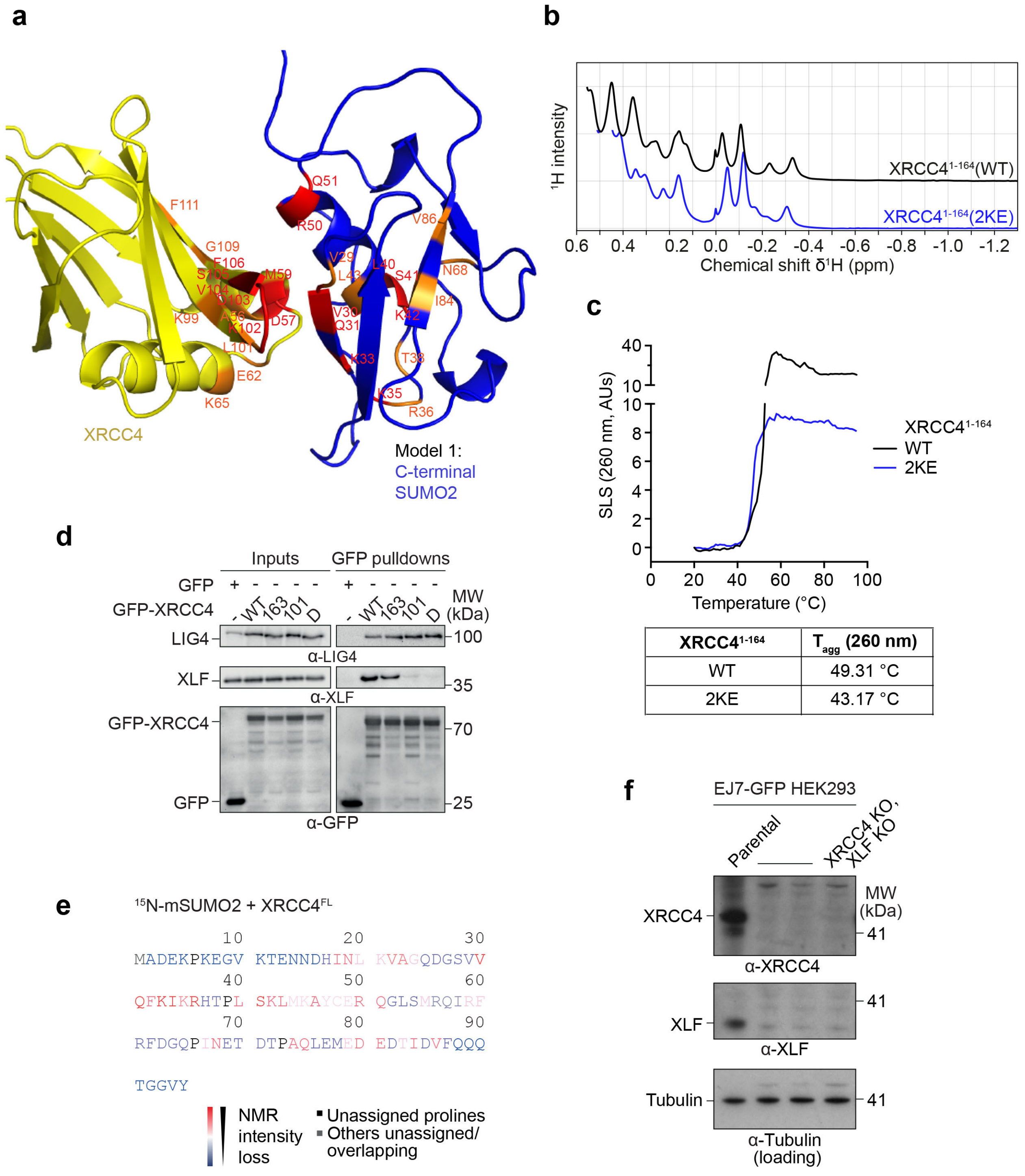
a
b
c
d
e
f
XRCC4
Model 1:
C-terminal
SUMO2
F111
G109
F106
S105
V104
D103
K102
L101
K99
A56
D57
M59
E62
K65
Q51
R50
V86
V29
L43
L40
S41
N68
I84
V30
Q31
K32
K33
T38
K35
R36
1H intensity
XRCC4 1-164(WT)
XRCC4 1-164(2KE)
0.6 0.4 0.2 0.0 -0.2 -0.4 -0.6 -0.8 -1.0 -1.2
Chemical shift δ1H (ppm)
SLS (260 nm, AUs)
Temperature (°C)
XRCC4 1-164
WT
2KE
XRCC4 1-164 | Tagg (260 nm)
WT | 49.31 °C
2KE | 43.17 °C
Inputs
GFP pulldowns
GFP + - - - - + - - - -
GFP-XRCC4 - WT 163 101 D - WT 163 101 D
MW (kDa)
LIG4
α-LIG4
100
XLF
α-XLF
35
GFP-XRCC4
70
GFP
25
α-GFP
15N-mSUMO2 + XRCC4FL
10 20 30
MADEKPKEGV KTENNDHINL KVAGQDGSVV
40 50 60
QFKIKRHTPL SKLMKAYCER QGLSMRQIRF
70 80 90
RFDGQPINET DTPAQLEMED EDTIDVFQQQ
TGGVY
NMR intensity loss
Unassigned prolines
Others unassigned/ overlapping
EJ7-GFP HEK293
Parental
XRCC4 KO, XLF KO
XRCC4
α-XRCC4
MW (kDa)
41
XLF
α-XLF
Tubulin
α-Tubulin (loading)

Supplementary Figure S7. Characterisation of SUMO2 binding to XRCC4, folding status of XRCC4$^{1-164}$ 2KE mutant and validation of EJ7-GFP XLF/XRCC4 double knock-out cell line. (**a**) Model 1 of 4xSUMO2-XRCC4 interactions, highlighting in red the interaction restraints used for HADDOCK “active” residues, and in orange for “passive” residues. Restraints were similarly adhered to in model 2. For details see Methods section. (**b**) 1D ^{1}H NMR spectra of wildtype (WT) XRCC4$^{1-164}$ and an XLF binding-deficient mutant (2KE; lysines 65 and 99 mutated to glutamic acids), showing the methyl regions of the spectra, which are extremely sensitive to structural perturbations, as the chemical shifts are defined by the angular and distance relationships with aromatic rings, which are fixed in the folded regions of proteins. Note that some resonances changed because methyl-containing residues have been substituted. XRCC4 WT spectrum replicated from Supplementary Figure S4a as reference. (**c**) Protein stability determination based on aggregation temperatures (T_{agg}’s) calculated from static light scattering (SLS) profiles measured over a defined temperature ramp as indicated. Note that SLS profiles after the start of aggregation can vary due to possible precipitation of the protein. XRCC4 WT profile replicated from Supplementary Figure S4b as reference. (**d**) Precipitation of LIG4 and XLF by GFP-Trap pulldowns of GFP-XRCC4 WT and SIM mutants, ectopically expressed in HEK293T cells. Pulldowns were performed twice with similar results. (**e**) Intensity losses in ^{1}H-^{15}N HSQC spectra of mSUMO2 after addition of increasing concentrations of full-length XRCC4 (XRCC4^{FL}). Colour gradient for XRCC4 residues ranges from red (most affected by binding) to blue (unaffected by binding). For details see Methods section. Unassigned prolines shown in black, other unassigned/overlapping residues in grey. (**f**) Validation of the HEK293 EJ7-GFP XLF KO, XRCC4 KO double knock-out cell line used for the EJ7-GFP assays (right), generated from parental HEK293 cells (left).
